# Precise Clearance of Intracellular MRSA via Internally and Externally Mediated Bioorthogonal Activation of Micro/Nano Hydrogel Microspheres

**DOI:** 10.1002/advs.202402370

**Published:** 2024-09-29

**Authors:** Jianye Yang, Li Chen, Zhengwei Cai, Libin Pang, Yanran Huang, Pengcheng Xiao, Juan Wang, Wei Huang, Wenguo Cui, Ning Hu

**Affiliations:** ^1^ Department of Orthopaedics The First Affiliated Hospital of Chongqing Medical University Orthopedic Laboratory of Chongqing Medical University Chongqing 400016 P. R. China; ^2^ Department of Orthopaedics Shanghai Key Laboratory for Prevention and Treatment of Bone and Joint Diseases Shanghai Institute of Traumatology and Orthopaedics Ruijin Hospital Shanghai Jiao Tong University School of Medicine 197 Ruijin 2nd Road Shanghai 200025 P. R. China

**Keywords:** bacillithiol response, bioorthogonal, copper nanocomplexes, hydrogel microspheres, sonodynamic therapy

## Abstract

Traditional high‐dose antibiotic treatments of intracellular methicillin‐resistant staphylococcus aureus (MRSA) are highly inefficient and associated with a high rate of infection relapse. As an effective antibacterial technology, sonodynamic therapy (SDT) may be able to break the dilemma. However, indiscriminate reactive oxygen species (ROS) release leads to potential side effects. This study incorporates Staphylococcal Protein A antibody‐modified Cu^2+^/tetracarboxyphenylporphyrin nanoparticles (Cu(II)NS‐SPA) into hydrogel microspheres (HAMA@Cu(II)NS‐SPA) to achieve precise eradication of intracellular bacteria. This eradication is under bioorthogonal activation mediated by bacillithiol (BSH) (internally) and ultrasound (US) (externally). To specify, the US responsiveness of Cu(II)NS‐SPA is restored when it is reduced to Cu(I)NS‐SPA by the BSH secreted characteristically by intracellular MRSA, thus forming a bioorthogonal activation with the external US, which confines ROS production within the infected M*Φ*. Under external US activation at 2 W cm^−2^, over 95% of intracellular MRSA can be cleared. In vivo, a single injection of HAMA@Cu(II)NS‐SPA achieves up to two weeks of antibacterial sonodynamic therapy, reducing pro‐inflammatory factor expression by 90%, and peri‐implant bone trabeculae numbers exceed the control group by five times. In summary, these micro/nano hydrogel microspheres mediated by internal and external bioorthogonal activation can precisely eliminate intracellular MRSA, effectively treating multi‐drug resistant intracellular bacterial infections.

## Graphical abstract

Cu(II)NS‐SPA, released by hydrogel microspheres, binds to the inherent Staphylococcal protein A on the surface of MRSA and is internalized into the cell through the modulating action of the antibody, further activates by BSH (internally) and US (externally) mediates bioorthogonal activation, and achieves clearance of intracellular MRSA. This approach offers a safe and effective solution to intracellular infections.

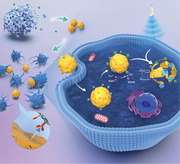

## 1 Introduction

Upon chemotactically migrating to the site of infection, macrophages (M*Φ*) engage in the specific recognition of bacteria. They play a pivotal role in the body's defense by engulfing and dismantling pathogens and then presenting their antigens to adaptive immune cells. This process ignites the innate immune response, constituting the primary line of defense against pathogenic invasions.^[^ ^1^ ^]^ However, burgeoning research indicates that Staphylococci, upon being engulfed by M*Φ*, are capable of establishing intracellular colonization. They achieve this through mechanisms like self‐induced dormancy, immune system modulation, secretion of protective elements, and the creation of an infectious microenvironment.^[^ ^2^ ^]^ In scenarios where treatment is halted or the host's immune function diminishes, these intracellular bacteria in M*Φ* can proliferate significantly, progressively wreaking havoc on host cells.^[^ ^3^ ^]^ This can result in dire infections, including osteomyelitis, sepsis, severe pneumonia, and endocarditis. Alarmingly, over two‐thirds of antibiotics currently available in clinical settings, vancomycin included, demonstrate negligible efficacy against these intracellular bacteria, especially within the safe thresholds of blood concentration.^[^ ^4^ ^]^ The fundamental issue lies in the fact that antibiotics need to traverse two cellular membranes to reach and act upon pathogens residing within phagolysosomes. The efficacy of antibiotics is significantly hampered by the lysosomes' highly acidic and hydrolytic environment, further compounding the challenge of effective antibacterial therapy.^[^ ^5^ ^]^ Therefore, traditional high‐dose antibiotic treatments are highly inefficient and associated with a high rate of infection relapse, severe systemic side effects, and the emergence of bacterial resistance. Consequently, developing effective therapeutic strategies to clear intracellular Staphylococci is of significant clinical importance for persistent/recurrent infections.

With advancements in biomaterial technology, there is an increasing emphasis among researchers on leveraging biomaterials as vectors for the targeted conveyance of antibiotics into M*Φ*, aimed at eradicating intracellular bacteria.^[^ ^6^ ^]^ This approach, while capable of altering the pharmacokinetics and tissue distribution of antibiotics in vivo to ensure preferential accumulation within M*Φ* and minimize systemic adverse effects.^[^ ^7^ ^]^ However, with the emergence of multiple drug‐resistant strains such as methicillin‐resistant staphylococcus aureus (MRSA) and vancomycin‐resistant Staphylococcus aureus, the effectiveness of antibiotics has drastically decreased, presenting new challenges in the eradication of intracellular bacteria.^[^ ^8^ ^]^ Reactive oxygen species (ROS) is considered to be one of the effective ways to kill multidrug‐resistant bacteria. Studies have shown that single‐atom iron nanozymes wrapped in macrophage membranes can produce highly toxic ROS in situ in the infected lesion to kill MRSA.^[^ ^7a^ ^]^ In addition, sonodynamic therapy (SDT) is an effective antibacterial technology that is effective in deep tissues. The large amount of ROS produced has a strong killing effect on almost all bacteria, avoiding the problem of drug resistance.^[^ ^9^ ^]^ Most sonosensitizers have low bioavailability and poor pharmacokinetics, often requiring repeated injections to obtain higher local concentrations. On the other hand, traditional sonosensitizers have poor cell permeability and lack cellular and/or bacterial targeting, making it difficult to clear MRSA hidden within macrophages even through repeated SDT treatment.^[^ ^10^ ^]^ The integration of nanomaterials with these traditional sonosensitizers can enhance their stability and target‐specificity, significantly boosting the antibacterial capabilities of SDT.^[^ ^11^ ^]^ Nevertheless, within these delivery methodologies, a majority of the drug compounds are metabolized and cleared by bodily tissues before they can effectively reach and impact the infection site, meaning only a minimal portion of the drugs can actually exert their intended biological effects.^[^ ^12^ ^]^ Additionally, these nano‐sonosensitizers struggle to align with the complex pathophysiology of intracellular infections, demonstrating macro‐level targeting in organs or tissues, but failing to precisely clear intracellular bacteria.^[^ ^13^ ^]^ On the other hand, existing SDT strategies for targeted antibacterial therapy, whether in physiological or pathological microenvironments, exhibit a constant ROS generation upon US stimulation (external), leading to indiscriminate tissue and cellular damage and wastage of sonosensitizers.^[^ ^14^ ^]^ Therefore, for antibacterial SDT against intracellular multi‐drug resistant bacterial infections, there is a need to design functionalized sonosensitizers. These should precisely deliver the drug intracellularly and confine US‐responsive ROS generation within infected M*Φ*, maximizing therapeutic effect while mitigating side effects.

Bioorthogonal reactions are strategies that necessitate specific pathological or physiological microenvironments to precisely control the delivery of bioactive molecules, achieving accurate targeted therapy.^[^ ^15^ ^]^ Studies have shown that Cu^2+^, with its unpaired electrons in a dual ground state, can effectively deactivate the US responsiveness of porphyrin‐based sonosensitizers upon chelation.^[^ ^16^ ^]^ When reduced to Cu^+^, the US responsiveness of the sonosensitizer can be reactivated. On the other hand, upon engulfment by M*Φ*, Staphylococcus aureus rapidly secrete bacillithiol (BSH), forming a characteristic intracellular reductive infection microenvironment.^[^ ^17^ ^]^ Thus, BSH can act as an effective internal activator for Cu^2+^‐chelated porphyrin sonosensitizers, forming a bioorthogonal activation mediated internally and externally with applied US, to achieve precise SDT. However, effectively delivering sonosensitizers into cells infected with MRSA remains a challenge to be addressed. Research indicates that using the conserved specific antigens on the surface of Staphylococcus aureus as molecular targets allows for the delivery of antibacterial agents into the cells, thereby effectively acting against intracellular MRSA.^[^ ^18^ ^]^ Therefore, integrating SPA‐specific antibodies into Cu^2+^‐chelated porphyrin sonosensitizers can effectively deliver this internally and externally mediated bioorthogonal activation strategy into infected M*Φ*. However, unbound nanoparticles are readily absorbed by bodily tissues and cleared by the immune system, necessitating high dosages and frequent injections to attain the desired therapeutic outcomes, thereby raising the risk of potential side effects.^[^ ^19^ ^]^ The development of injectable hydrogel microspheres, fabricated utilizing microfluidic technology, offers a promising solution to this issue.^[^ ^20^ ^]^ These carriers can significantly enhance the local retention of nanoparticles, optimizing their biological efficacy and minimizing systemic exposure.^[^ ^21^ ^]^

Therefore, we aim to use SDT to break through the clinical treatment dilemma of intracellular infection, further improve the efficacy of traditional SDT, and reduce side effects. In this study, we propose a therapeutic strategy that targets intracellular MRSA and precisely releases ROS to clear intracellular bacteria under bioorthogonal activation formed by the infection microenvironment (internally) and US (externally). Initially, we utilized Cu^2+^‐chelated with tetracarboxyphenylporphyrin to deactivate its US responsiveness and introduced SPA via the Michael addition reaction, obtaining functionalized nanoparticles, namely Cu(II)NS‐SPA. We then integrated Cu(II)NS‐SPA into methacrylated hyaluronic acid (HAMA) hydrogel microspheres (HAMA@Cu(II)NS‐SPA) using microfluidic technology. According to the experimental hypothesis, HAMA@Cu(II)NS‐SPA binds to the inherent Staphylococcal protein A on the surface of MRSA and is internalized into the cell through the modulating action of the antibody, further activated by BSH (internally) and US (externally) mediated bioorthogonal activation, and achieves clearance of intracellular MRSA. In vitro results show that Cu(II)NS‐SPA could clear over 95% of intracellular MRSA under internally and externally mediated bioorthogonal activation. Additionally, HAMA@Cu(II)NS‐SPA showed a high retention rate and could stably release Cu(II)NS‐SPA over two weeks. In a rat periprosthetic joint infection (PJI) model, HAMA@Cu(II)NS‐SPA effectively cleared MRSA, thereby reducing over 90% of pro‐inflammatory factor expression in the lesion area, and promoting bone integration. In summary, we established an intracellular precision SDT strategy based on micro/nano composite microspheres mediated by internal and external bioorthogonal activation, achieving on‐demand clearance of intracellular bacteria. The treatment of multidrug‐resistant bacteria‐related intracellular infections could be safe and clinically promising with this approach.

## 2 Results and Discussion

### 2.1 Preparation and Characterization of Cu(II)NS‐SPA

**Scheme 1:**
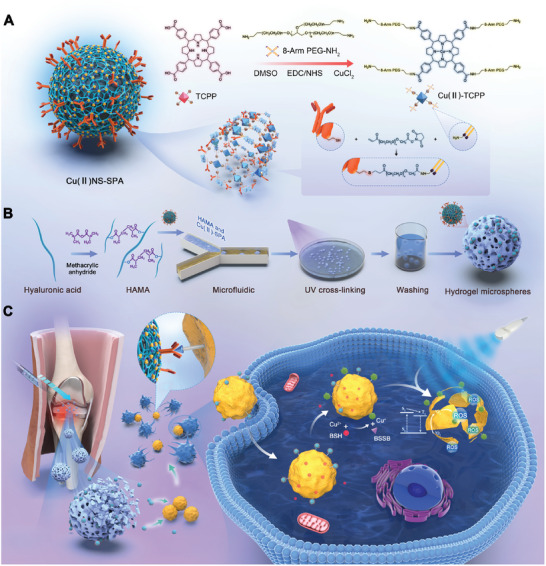
The design, medication, and biological orthogonal activation mechanism of HAMA@Cu(II)NS‐SPA. A) The comprehensive construction process of Cu(II)NS‐SPA. B) The procedure for constructing HAMA@Cu(II)NS‐SPA hydrogel microspheres containing Cu(II)NS‐SPA. C) Through bioorthogonal activation mediated internally by BSH secreted by intracellular MRSA and externally by ultrasound, HAMA@Cu(II)NS‐SPA precisely releases ROS to eradicate intracellular bacteria.

**Figure 1:**
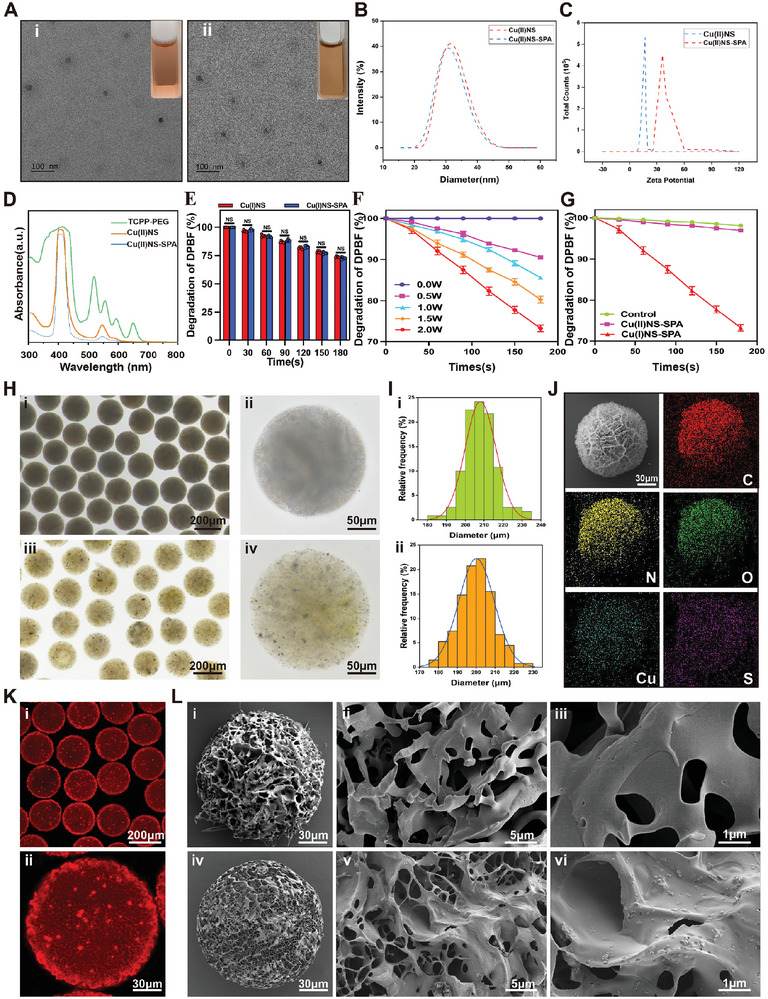
Material characterization of bioorthogonally activated micro/nano hydrogel microspheres mediated internally and externally. A) Representative SEM images of Cu(II)NS (i) and Cu(II)NS‐SPA (ii), with the top‐left corner showing a photograph of the nanoparticle solution. B) The size distribution of Cu(II)NS and Cu(II)NS‐SPA. C) The zeta potential of Cu(II)NS and Cu(II)NS‐SPA. D) UV–vis spectra of TCPP‐PEG, Cu(II)NS, and Cu(II)NS‐SPA. E) ROS production rate of Cu(I)NS and Cu(I)NS‐SPA (2 W cm^−2^, 1 MHz, 50% duty cycle, 2 min). F) ROS production of Cu(I)NS‐SPA under different ultrasound intensities (1 MHz, 50% duty cycle) and treatment durations. G) Changes in ROS production over time for deionized water, Cu(I)NS‐SPA, and Cu(II)NS‐SPA under ultrasound treatment (2 W cm^−2^, 1 MHz, 50% duty cycle). The t‐test results showed that Cu(I)NS‐SPA can generate ROS and consume a large amount of DPBF, and there was no statistical difference in the degradation of DPBF between the Control group and the Cu(II) NS group. H) Bright field images of HAMA (i, ii) and HAMA@Cu(II)NS‐SPA (iii, iv). I) Particle size distribution of HAMA (i) and HAMA@Cu(II)NS‐SPA (ii). J) Elemental mapping of HAMA@Cu(II)NS‐SPA. K) LSCM images of HAMA@Cu(II)NS‐SPA. L) SEM images of HAMA (i‐iii) and HAMA@Cu(II)NS‐SPA (iv‐vi). (*p < 0.05, **p < 0.01, ***p < 0.001, ****p < 0.0001; data are expressed as mean ± standard deviation, *n *= 5 independent experiments, student‐*t* test, and One – way ANOVA).

As shown in **Scheme** **1**A, we first modified TCPP with 8‐arm‐PEG‐NH_2_, then chelated with Cu^2+^ to produce Cu(II)NS. To confer bacterial targeting and enhance cellular uptake of Cu(II)NS, we acryloylated it using N‐acryloxysuccinimide (NHS‐Ac), followed by a Michael addition reaction with SPA containing free thiol groups, obtaining Cu(II)NS‐SPA. Afterward, we examined Cu(II)NS versus Cu(II)NS‐SPA. Images taken with transmission electron microscopy (TEM) (**Figure** **1**A) show ≈30 nm‐sized Cu(II)NS particles, whereas Cu(II)NS‐SPA, similar in morphology to Cu(II)NS, has a slightly increased size of ≈32 nm, likely due to SPA surface modification. Dynamic light scattering measurements are consistent with those obtained by TEM (Figure 1B). As shown in Figure 1C, Cu(II)NS has a positive Zeta potential of 16.8 ± 0.5 mV, while Cu(II)NS‐SPA exhibits a higher positive Zeta potential of 36.8 ± 2.5 mV. Studies indicate that the surface of MRSA is negatively charged. Therefore, SPA modification not only allows specific antigen‐antibody binding with MRSA but also enhances the electrostatic attraction between the nanoparticles and MRSA, improving the targeting efficiency of the nanoparticles to MRSA. Thus, Cu(II)NS‐SPA represents an efficient strategy for targeting MRSA through internally and externally mediated bioorthogonal activation for SDT. Furthermore, we examined the UV absorption spectra of Cu(II)NS‐SPA, Cu(II)NS, and TCPP‐Cu (Figure 1D). The presence of inequivalent N atoms in TCPP‐PEG results in four peaks between 500–700 nm. After chelation with Cu^2+^, which forms coordination with the N atoms, only one peak is observed. On the other hand, the SPA modification does not significantly affect the UV absorption peak of the nanoparticles. We further investigated the reaction efficiency of Cu(II)NS‐SPA and Cu(II)NS with BSH, finding that BSH effectively reduces Cu^2+^ to Cu^+^, and the SPA modification does not impact the reaction between Cu2+ and BSH (Figure 1E).

In contrast to Cu(II)NS‐SPA, Cu(I)NS‐SPA exhibits high sonosensitivity due to its paired electrons. The ROS generation of Cu(II)NS requires bioorthogonal activation formed by internal BSH and external US. In the beginning, we mixed a solution of Cu(II)NS‐SPA (10 × 10^−3^ m, 1 mL) with a BSH solution (10 × 10^−3^ m, 2 mL) and incubated it in the dark at 37 °C with shaking for 2 h to obtain Cu(I)NS‐SPA. Cu(I)NS was prepared in a similar way. And we found that BSH effectively reduces Cu^2+^ to Cu^+^. X‐ray photoelectron spectroscopy of Cu 2p shows that the ratio of Cu(II)/Cu(I) in Cu(II)NS is 0.692 (Figure S1A,B, Supporting Information). The existence of Cu(I) in Cu(II)NS can be due to the coordination bond of Cu─N. While the ratio of Cu(II)/Cu(I) is 0.213 in the obtained Cu(I)NS (Figure S1A,C, Supporting Information), indicating that BSH could reduce Cu(II) to Cu(I). And the SPA modification does not impact the reaction between Cu^2+^ and BSH (Figure S2, Supporting Information). We evaluated the singlet oxygen (^1^O_2_) production by Cu(I)NS‐SPA and Cu(I)NS at various time points, using the degradation of 1,3‐diphenylisobenzofuran (DPBF) as an indicator, under consistent US power. The ^1^O_2_ generation rates of Cu(I)NS‐SPA and Cu(I)NS demonstrated no significant statistical disparity as anticipated, confirming that SPA modification does not adversely impact the SDT efficacy (Figure 1E). Figure 1F illustrates that the ^1^O_2_ production rate of Cu(I)NS‐SPA is directly proportional to the US power and the duration of treatment. Following this, we assessed the ^1^O_2_ generation rates of Cu(I)NS‐SPA and Cu(II)NS‐SPA at various intervals under identical US conditions. The DPBF degradation in the Cu(II)NS‐SPA samples showed no notable statistical deviation from the control group and was markedly lower than that in the Cu(I)NS‐SPA group (1G). These findings underscore that Cu^2+^ efficiently nullifies the sonosensitivity of Cu(II)NS‐SPA, and the targeted internal activation by BSH is a crucial element for precise SDT. Overall, Cu(II)NS‐SPA emerges as an agent adept at specifically targeting MRSA, adeptly releasing ROS in response to bioorthogonal activation induced by BSH, which is selectively secreted by intracellular MRSA, and externally complemented by the US.

### 2.2 Construction and Characterization of HAMA@Cu(II)NS‐SPA

In order to decrease tissue absorption and immune clearance of Cu(II)NS‐SPA, as well as the frequency of intraarticular injections, we employed microfluidic and photopolymerization technologies to construct injectable hydrogel microspheres as carriers (Scheme 1B), providing continuous, stable, and precise SDT to the infected area. Hyaluronic acid (HA), a component of the extracellular matrix, known for its excellent biocompatibility, has been used by our group to develop various promising injectable carriers.^[^ ^20^ ^]^ At the outset, HA was modified with methacrylic anhydride to introduce photocurable carbon–carbon double bonds, yielding HAMA. Proton nuclear magnetic resonance spectroscopy confirmed the successful introduction of double bonds, with a grafting rate of ≈83.5% (Figure S3, Supporting Information). Afterward, we used microfluidic technology to generate pre‐gel droplets of HAMA and HAMA@Cu(II)NS‐SPA (Figure S4, Supporting Information), which were further crosslinked with UV light to form hydrogel microspheres. As shown by optical microscopy, HAMA@Cu(II)NS‐SPA composite microspheres exhibit similar good dispersion, uniform shape, and size as HAMA microspheres (Figure 1H). The average diameter of HAMA microspheres was 208 µm, and that of HAMA@Cu(II)NS‐SPA composite microspheres was 200 µm (Figure 1I), with no significant difference between them. To confirm the successful loading of Cu(II)NS‐SPA into the hydrogel microspheres, we labeled SPA with Cy3 and observed it using confocal microscopy. The results (Figure 1K) show that nanoparticles for internally and externally mediated bioorthogonal activation were successfully integrated into the hydrogel microspheres. Scanning electron microscopy (SEM) revealed the microstructure of the hydrogel microspheres (Figure 1L), indicating that HAMA@Cu(II)NS‐SPA possesses a porous structure conducive to sustained and controlled release of nanoparticles, and demonstrates high loading efficiency for Cu(II)NS‐SPA. To assess the distribution of Cu(II)NS‐SPA within the microspheres, we employed energy‐dispersive X‐ray spectroscopy to evaluate the elemental distribution (Figure 1J), indicating the uniform distribution of Cu and S (from SPA) within the microspheres. It is crucial for the carriers of Cu(II)NS‐SPA to have appropriate biodegradability and sustained release properties. If the carrier degrades too quickly, long‐term SDT treatment cannot be achieved, while too slow degradation may hinder subsequent tissue repair. As shown in Figure S5 (Supporting Information), the microspheres undergo rapid degradation in the first two weeks, releasing over 90% of Cu(II)NS‐SPA. This degradation and release behavior over two weeks is suitable for the treatment in PJI animal models. Therefore, the hydrogel microspheres as carriers for internally and externally mediated bioorthogonal activation for precise SDT can effectively reduce intra‐articular injections, achieving continuous SDT treatment over two weeks.

### 2.3 In Vitro Antibacterial Performance Evaluation

**Figure 2:**
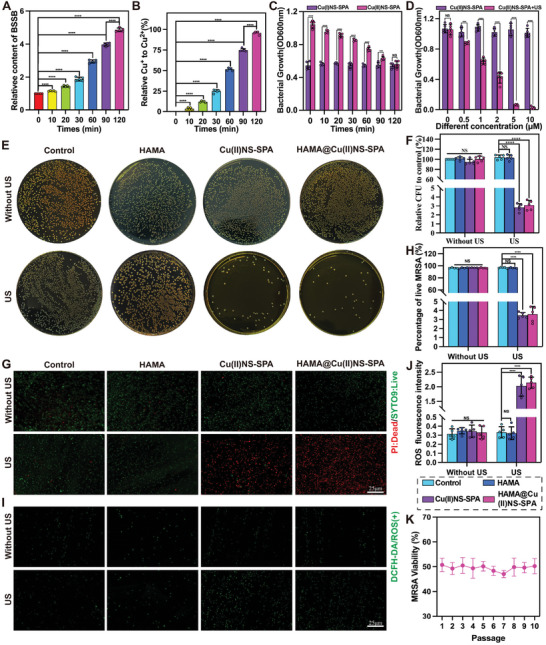
In vitro antimicrobial efficacy and drug resistance assessment of bioorthogonally activated micro/nano hydrogel microspheres mediated internally and externally. A) Relative concentration of BSSB in bacterial solution after incubation of Cu(II)NS‐SPA with intracellular MRSA. B) Relative concentration of Cu(I)NS‐SPA after incubation with intracellular MRSA and Cu(II)NS‐SPA. C) Influence of Cu(II)NS‐SPA and Cu(I)NS‐SPA on bacterial growth during different incubation times. D) Impact of ultrasound treatment on MRSA growth after incubation with Cu(II)NS‐SPA. E) Representative images of standard colony counts following various treatments. F) Quantitative analysis of bacterial colonies. G) Representative images of bacterial live/dead staining. H) Quantitative analysis of live/dead staining. I) Generation of bacterial ROS following various treatments. J) Quantitative analysis of ROS generation. K) Changes in MRSA drug resistance following continuous exposure to biorthogonally activated SDT. f no special instructions or markings are given, the ultrasound parameters are 2 W cm^−2^, 1 MHz, 50% duty cycle, 2 min. (*p < 0.05, **p < 0.01, ***p < 0.001, ****p < 0.0001; data are expressed as mean ± standard deviation, *n* = 5 independent experiments, student*‐t* test, and One – way ANOVA).

To evaluate the influence of ROS generated by nanoparticles under US stimulation on bacterial growth, we executed a minimum inhibitory concentration (MIC) test. Starting with, to eliminate the direct effects of the US on bacteria, their viability was examined under various US powers and durations. Figure S6 (Supporting Information) illustrates that the US exerted a minimal impact on bacterial proliferation. Moving forward, MRSA cultures were treated with different concentrations of Cu(I)NS‐SPA and Cu(II)NS‐SPA, followed by US exposure (2 W cm^−2^, 1 MHz, 50% duty cycle, 120 s). Spectrophotometric analysis indicated that a mere 0.5 µm concentration of Cu(I)NS‐SPA notably hindered bacterial growth, with 10 µm suppressing over 95% of it (as depicted in Figure S7, Supporting Information). Conversely, Cu(II)NS‐SPA under US treatment showed no substantial inhibition of bacterial growth. It's worth mentioning, as previously highlighted, that Cu^2+^ effectively disables the ROS‐generating capacity of Cu(II)NS‐SPA. We used MRSA to infect M*Φ* and isolated intracellular surviving strains, and there is a high‐secretion state of BSH in these MRSA strains. We incubated Cu(II)NS‐SPA with normal MRSA or intracellular MRS, measuring the levels of BSH oxidation product BSSB and Cu^+^ to assess the specific internal activation by BSH. The results showed that, over time, the intracellular MRSA group exhibited a gradual increase in BSSB and Cu(I)NS‐SPA content (**Figure** **2**A,B). This indicates that BSH, abundantly secreted by intracellular MRSA, is a specific activator for the US responsiveness of Cu(II)NS‐SPA. To further confirm this hypothesis, we incubated Cu(II)NS‐SPA with normal MRSA and intracellular MRSA (internally) and then applied US treatment (1 MHz, 50% duty cycle, 120 s) externally. The internally and externally mediated bioorthogonal activation exhibited more potent bactericidal capabilities. With increasing incubation time, the Cu(II)NS‐SPA group showed enhanced bactericidal activity, and at 2 h of incubation, the efficiency was comparable to the direct use of Cu(I)NS‐SPA (Figure 2C). Additionally, we compared the effects of different concentrations of Cu(II)NS‐SPA on bacterial growth, with and without US treatment, after 2 h of incubation with bacteria. As shown in Figure 2D, without the US, Cu(II)NS‐SPA had almost no effect on bacterial growth. However, under US stimulation, 10 µM of Cu(II)NS‐SPA killed over 95% of bacteria. Subsequently, we evaluated the antibacterial activity of HAMA@Cu(II)NS‐SPA against MRSA using standard colony counting methods. The results indicated that without external US stimulation, all treatments showed no significant bactericidal activity (Figure 2E,F). Post‐US treatment, both HAMA@Cu(II)NS‐SPA extract and Cu(II)NS‐SPA demonstrated significant bactericidal effects against MRSA. As depicted in Figure 2G,H, most samples were stained green with STOY9, while a large number of dead bacteria, stained red with propidium iodide, were found in the US‐treated HAMA@Cu(II)NS‐SPA and Cu(II)NS‐SPA groups. These findings suggest that the bioorthogonal activation‐mediated micro/nano hydrogel microspheres display potent bactericidal activity against MRSA. As mentioned earlier, SDT primarily exerts its bactericidal effects through the generation of ROS. Therefore, we further detected ROS production in the bacterial solution post‐US treatment. As shown in Figure 2I,J, in the absence of US treatment, ROS positivity rates were low in all groups. Nonetheless, both HAMA@Cu(II)NS‐SPA and Cu(II)NS‐SPA released substantial ROS for bactericidal purposes under US treatment. It is well‐known that bacteria continually evolve drug resistance, posing significant challenges in clinical treatment. Hence, we further evaluated the potential for MRSA to develop resistance following SDT treatment. Encouragingly, the robust bactericidal performance of HAMA@Cu(II)NS‐SPA maintained its full strength even after ten consecutive passages, as demonstrated in Figure 2K. This confirms that the strategy of internally and externally mediated bioorthogonal activation micro/nano hydrogel microspheres can effectively mitigate the evolution of drug‐resistant bacteria in antimicrobial treatments. In summary, HAMA@Cu(II)NS‐SPA exhibits strong bactericidal capabilities and can effectively prevent the development of MRSA resistance.

**Figure 3:**
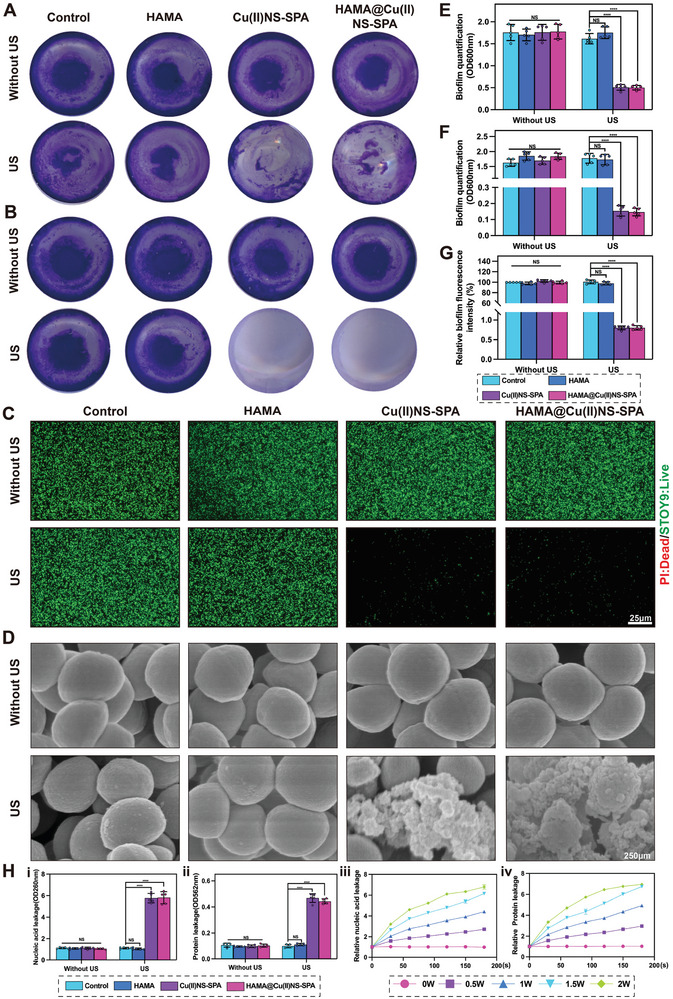
The effect of internally and externally mediated bioorthogonal activation of micro/nano hydrogel microspheres on biofilm formation and ROS antibacterial mechanism. A) The inhibitory effect of different treatments on biofilm formation. B) The disruptive effect of different treatments on biofilms. C) LSCM observation of the inhibitory effects on biofilm formation by different treatments. D) Representative SEM images of bacterial biofilm disruption under different treatments. E) Quantitative analysis of biofilm formation inhibition by measuring crystal violet content. F) Quantitative analysis of biofilm disruption by measuring crystal violet content. G) Fluorescent quantitative analysis of biofilms. H) The effect of different treatments on bacterial nucleic acid and protein leakage. f no special instructions or markings are given, the ultrasound parameters are 2 W cm^−2^, 1 MHz, 50% duty cycle, 2 min. (*p < 0.05, **p < 0.01, ***p < 0.001, ****p < 0.0001; data are expressed as mean ± standard deviation, *n *= 5 independent experiments, student‐t test, and One – way ANOVA).

In cases of PJI, the development of biofilms around metal implants is pivotal in facilitating MRSA's evasion of immune responses and resistance to antibiotic treatments.^[^ ^22^ ^]^ Thus, we delved into examining the effects of significant ROS release from HAMA@Cu(II)NS‐SPA on both the formation and disruption of MRSA biofilms. As depicted in **Figure** **3**A and corroborated by the quantitative data in 3E, exposure to Cu(II)NS‐SPA and HAMA@Cu(II)NS‐SPA under ultrasound effectively thwarted biofilm formation on implant surfaces. Further investigations revealed that, under ultrasound intervention, both HAMA@Cu(II)NS‐SPA and Cu(II)NS‐SPA could dismantle existing biofilms, thereby facilitating the effective removal of bacteria from implants (Figure 3B,F). This outcome was further substantiated by fluorescent staining analyses of the biofilms (as illustrated in Figure 3C,G). Overall, HAMA@Cu(II)NS‐SPA plays a critical role in obstructing and disintegrating MRSA biofilms, significantly boosting the clinical efficacy in treating PJI by dismantling MRSA's protective barrier on implant surfaces.

As previously mentioned, HAMA@Cu(II)NS‐SPA effectively kills MRSA and disrupts biofilms by releasing substantial ROS under US stimulation. We delved further into the mechanism of ROS‐mediated MRSA eradication. The integrity of bacterial membranes is vital for MRSA survival; its disruption leads to the leakage of bacterial contents, ultimately resulting in death. Initially, we observed MRSA post‐treatment using SEM. As shown in Figure 3D, Cu(II)NS‐SPA and HAMA@Cu(II)NS‐SPA under US stimulation caused bacterial collapse and even fragmentation, indicating that this internally and externally mediated bioorthogonal activation strategy effectively disrupts bacterial membrane structure, leading to MRSA death. Next, we employed cell leakage tests to confirm this phenomenon by detecting proteins and nucleic acids released by bacteria. In the initial phase, we eliminated the influence of US treatment of varying intensities and durations on the cell leakage test (Figure S8, Supporting Information). Then, we assessed the impact of Cu(II)NS‐SPA on cell leakage under different US intensities and durations. As depicted in Figure 3H, the leakage of proteins and nucleic acids caused by Cu(II)NS‐SPA increased with the US power and treatment duration. In conclusion, our experimental results indicate that HAMA@Cu(II)NS‐SPA disrupts bacterial membrane integrity by generating ROS, leading to bacterial fragmentation. This observation could partially elucidate the underlying mechanism of the potent bactericidal efficacy inherent in the bioorthogonal activation strategy of SDT, mediated both internally and externally.

### 2.4 In Vitro Biocompatibility Evaluation

**Figure 4:**
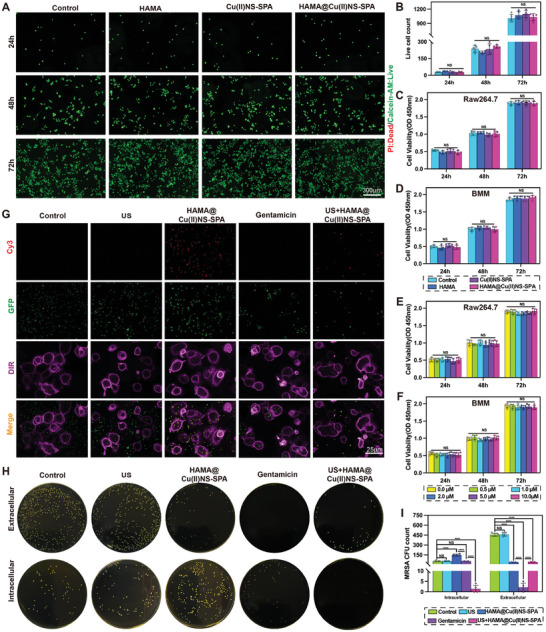
Cellular biocompatibility and intracellular bactericidal results of biorthogonally activated micro/nano hydrogel microspheres mediated internally and externally. A) Representative images of live/dead cell staining under different treatments. B) Quantitative analysis results of live/dead cell counts. C–F) CCK‐8 assay results of RAW264.7 and BMM cells under different treatments. G) The clearance effect of HAMA@Cu(II)NS‐SPA and gentamicin on intracellular and extracellular bacteria, and enhancement of macrophage phagocytosis by nanoparticles. H) Representative images of standard colony counts for intracellular and extracellular MRSA after various treatments. I) Quantitative analysis of plate bacterial counts. *f* no special instructions or markings are given, the ultrasound parameters are 2 W cm^−2^, 1 MHz, 50% duty cycle, 2 min. (*p < 0.05, **p < 0.01, ***p < 0.001, ****p < 0.0001; data are expressed as mean ± standard deviation, *n* = 5 independent experiments, student‐*t* test, and One – way ANOVA).

As a potential strategy for PJI treatment, HAMA@Cu(II)NS‐SPA should exhibit good biocompatibility. By using live/dead staining and the CCK‐8 assay, we evaluated the effect of this therapeutic system on the proliferation and viability of RAW264.7 cells and bone marrow‐derived macrophages (BMMs). As shown in **Figure** **4**A,B, the cells grew well during the 72‐hour co‐culture period, with almost no cell death observed. Consistent with the live/dead staining results, the numbers of RAW264.37 (Figure 4C,E) and BMMs (Figure 4D,F) cells increased over time, and the cell viability in HAMA, Cu(II)NS‐SPA, and HAMA@Cu(II)NS‐SPA groups showed no significant difference compared to the control group. Additionally, even at concentrations as high as 10 µM, Cu(II)NS‐SPA did not affect the cell viability of RAW264.37 and BMMs. These results indicate that HAMA@Cu(II)NS‐SPA itself does not impair the viability of M*Φ* and possesses good biocompatibility, making it suitable for precise intracellular treatment of PJI.

### 2.5 Intracellular Bactericidal Performance of MΦ

Intracellular pathogen infection is one of the primary causes of persistent and recurrent PJI in clinical settings. To address this, we proposed a therapeutic strategy that targets intracellular MRSA and precisely releases ROS under bioorthogonal activation formed by the infection microenvironment (internally) and US (externally). Commencing with, we evaluated the ability of antibiotics and HAMA@Cu(II)NS‐SPA to clear intracellular MRSA. As shown in Figure 4G, gentamicin effectively killed extracellular MRSA (green) but failed to clear intracellular pathogens. In contrast, HAMA@Cu(II)NS‐SPA (red) could target MRSA, disrupt the redox balance caused by intracellular BSH, and effectively clear intracellular pathogens under US activation, while extracellular Cu(II)‐SPA remained unactivated and thus did not clear MRSA. Additionally, after MRSA bound to Cu(II)‐SPA, phagocytosis by M*Φ* significantly increased. We performed standard colony counting of both extracellular and intracellular MRSA thereafter to accurately assess the bactericidal effects of gentamicin and HAMA@Cu(II)NS‐SPA (Figure 4H,I). Consistent with the immunofluorescence results, the Cu(II)NS‐SPA group had a significantly lower intracellular MRSA colony count than the control or gentamicin groups. The number of intracellular bacteria increased in the Cu(II)NS‐SPA group, suggesting that SPA can target‐bind with MRSA and promote the phagocytosis of pathogens by M*Φ* through opsonization. This suggests that the internally and externally mediated bioorthogonal activation SDT strategy can effectively clear MRSA within M*Φ*, mitigating the pathogen's immune evasion effect. In summary, our results demonstrate that HAMA@Cu(II)NS‐SPA, through functional antibody SPA's specific binding to SasA, can target MRSA and promote phagocytosis by M*Φ*. It further precisely clears intracellular MRSA under bioorthogonal activation formed by BSH (internally) and US (externally). Overall, HAMA@Cu(II)NS‐SPA represents a highly promising treatment strategy for PJI.

### 2.6 Efficacy Evaluation in a Rat PJI Model

**Figure 5:**
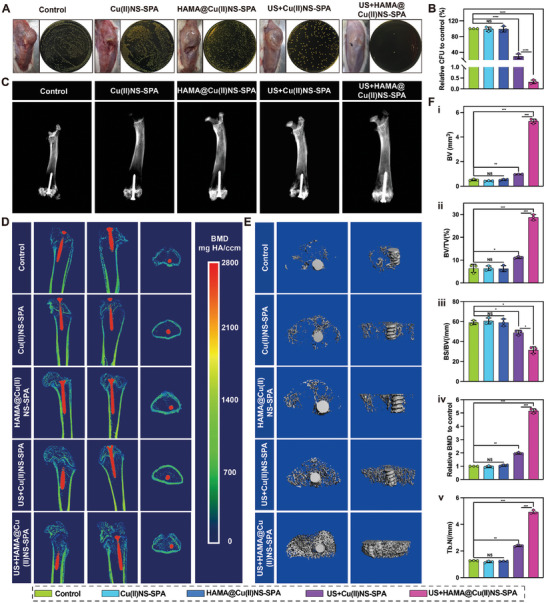
Imaging evaluation of the in vivo therapeutic efficacy of biorthogonally activated micro/nano hydrogel microspheres mediated internally and externally. A) Macroscopic images of rat knee joints in different experimental groups and standard colony count plates of tissue around the prosthesis. B) Quantitative analysis results of plate bacterial counts. C) X‐ray films of the distal femur of rats. D) MicroCT images of the distal femur of rats. E) Representative 3D reconstructed images of trabeculae around the femoral prosthesis in rats. F) Quantitative analysis of bone volume (BV), bone volume fraction (BV/TV), bone mineral density (BMD), number of trabeculae (Tb.N), and bone surface area/bone volume. (*p < 0.05, **p < 0.01, ***p < 0.001, ****p < 0.0001; data are expressed as mean ± standard deviation, *n *= 3 independent experiments, student‐*t* test, and One – way ANOVA).

We established a rat PJI model to evaluate its in vivo anti‐infective efficacy given the potent in vitro bactericidal effects of HAMA@Cu(II)NS‐SPA. Two weeks post‐infection, the control, the Cu(II)NS‐SPA, and the HAMA@Cu(II)NS‐SPA group exhibited significant abscesses in knee joint tissues (**Figure** **5**A). The US+Cu(II)NS‐SPA group showed reduced abscesses compared to the control group, while the US+HAMA@Cu(II)NS‐SPA group displayed almost no abscesses or inflammatory edema, indicating excellent anti‐infection efficacy. Subsequently, we ground the tissues around the implant for standard colony counting. The results, as depicted in Figure 5A,B, showed almost no bacterial growth in the US+HAMA@Cu(II)NS‐SPA group, while US+Cu(II)NS‐SPA could reduce the bacterial load around the implant to some extent, but the effect was not ideal. Overall, Cu(II)NS‐SPA under US exposure demonstrated some antibacterial effect, and HAMA significantly enhanced its in vivo efficacy, achieving precise and efficient treatment of PJI.

Periprosthetic osteolysis is one of the most catastrophic consequences of PJI, as it can lead to implant loosening and render it unusable.^[^ ^23^ ^]^ To start off, we evaluated periprosthetic bone resorption using X‐ray imaging. As shown in Figure 5C, the control, Cu(II)NS‐SPA, and HAMA@Cu(II)NS‐SPA group showed a noticeable decrease in bone density around the implant, whereas the US+HAMA@Cu(II)NS‐SPA group exhibited the highest bone density. To more accurately assess periprosthetic bone resorption, we further utilized Micro‐CT to analyze microstructural changes and bone remodeling at the distal femur of the animal model. As illustrated in Figure 5D,E, the US+HAMA@Cu(II)NS‐SPA group had higher bone volume, and there were significantly more trabeculae than in the control group around the implant. Quantitative analysis revealed that the US+HAMA@Cu(II)NS‐SPA treatment group had higher bone volume (BV), bone volume fraction (BV/TV), trabecular number (Tb.N), and bone mineral density (BMD), as well as a lower bone surface area to bone volume ratio (Figure 5F). Overall, US+HAMA@Cu(II)NS‐SPA markedly improved periprosthetic bone resorption.

**Figure 6:**
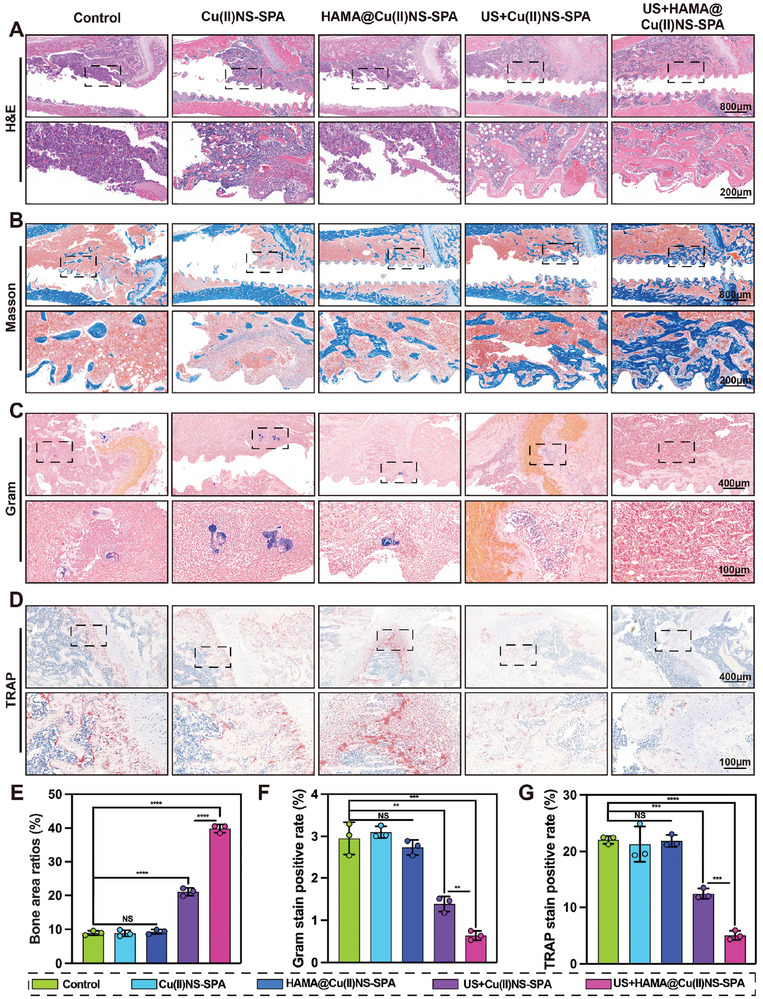
Histological evaluation of the in vivo therapeutic efficacy of biorthogonally activated micro/nano hydrogel microspheres mediated internally and externally. A) Representative images of HE staining. B) Representative images of Masson's trichrome staining. C) Representative images of Gram staining. D) Representative images of TRAP staining. E) Quantitative analysis of new bone formation around the prosthesis in Masson's staining. F) Quantitative analysis of Gram staining. G) Quantitative analysis of aberrant activation of osteoclasts around the prosthesis. (*p < 0.05, **p < 0.01, ***p < 0.001, ****p < 0.0001; data are expressed as mean ± standard deviation, *n* = 3 independent experiments, student*‐t* test, and One – way ANOVA).

**Figure 7:**
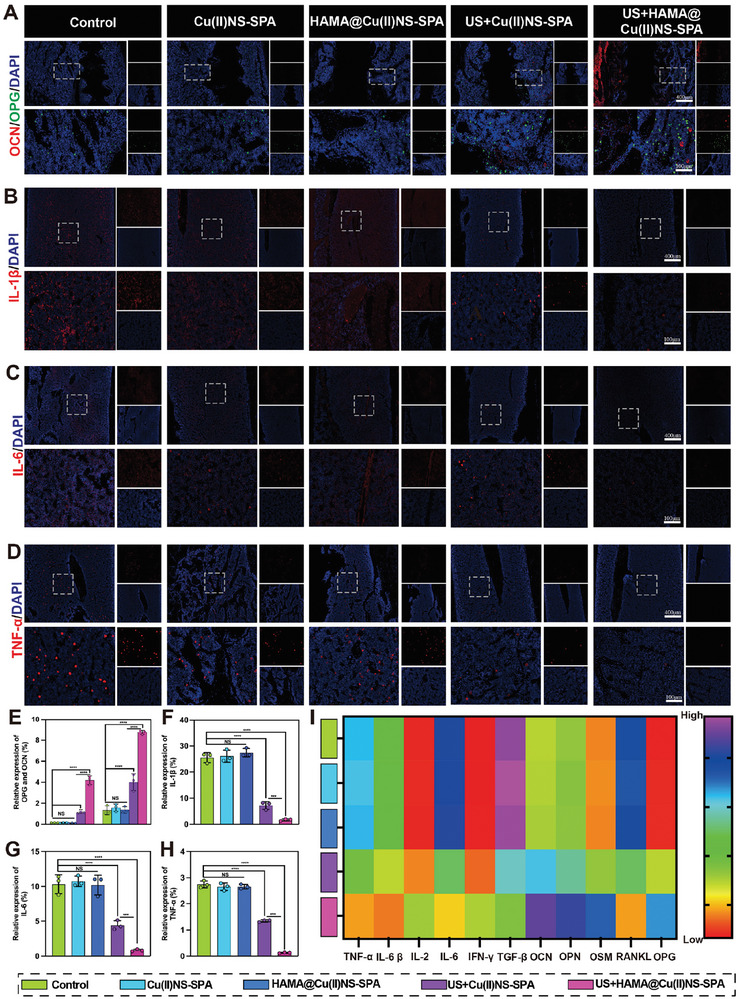
The role of internally and externally mediated biorthogonally activated micro/nano hydrogel microspheres in inflammation control and bone remodeling. A) Representative immunofluorescent staining images of OCN and OPG. B–D) Representative immunofluorescent staining images of pro‐inflammatory factors IL‐1β, IL‐6, TNF‐α. E) Quantitative analysis of the relative fluorescence intensity of OCN and OPG. F–H) Quantitative analysis of the relative fluorescence intensity of pro‐inflammatory factors IL‐1β, IL‐6, TNF‐α using Image J software. I) Heatmap of the expression of proinflammatory factors, osteogenic markers, and osteoblastic markers in periprosthetic tissues. (*p < 0.05, **p < 0.01, ***p < 0.001, ****p < 0.0001; data are expressed as mean ± standard deviation, *n* = 3 independent experiments, student‐*t* test, and One – way ANOVA).

We employed histological staining analysis as the next step to evaluate the in vivo therapeutic effects of US+HAMA@Cu(II)NS‐SPA. In the beginning, H&E staining (**Figure** **6**A) revealed severe bone destruction and extensive inflammatory cell infiltration in the control, Cu(II)NS‐SPA, and HAMA@Cu(II)NS‐SPA group, indicative of typical bone infection. Similar to previous results, US+Cu(II)NS‐SPA showed some therapeutic effects but was not as effective as the US+HAMA@Cu(II)NS‐SPA group. Masson's trichrome staining (Figure 6B) and its quantitative results (**Figure** **7**E) suggested that US+HAMA@Cu(II)NS‐SPA had the most new bone formation, demonstrating its efficacy in treating PJI and preventing periprosthetic loosening. Moreover, Gram staining results (Figure 6C,F) were consistent with earlier standard colony counting outcomes, showing significant bacterial residue in the control, Cu(II)NS‐SPA, and HAMA@Cu(II)NS‐SPA groups. In contrast, the US+HAMA@Cu(II)NS‐SPA group had virtually no residual bacteria. Last, TRAP staining (Figure 6D) and its quantitative analysis (Figure 6G) indicated that bacterial infection led to the activation of numerous osteoclasts around the implant, promoting bone resorption. The US+HAMA@Cu(II)NS‐SPA group, through its potent antibacterial effect, effectively mitigated local inflammation and inhibited the abnormal activation of periprosthetic osteoclasts, thereby reducing bone resorption. In conclusion, US+HAMA@Cu(II)NS‐SPA represents a highly promising therapeutic strategy for PJI.

The catastrophic outcomes such as bone resorption in PJI are fundamentally due to a persistent inflammatory response during infection, leading to an imbalance in bone homeostasis mediated by osteoblasts and osteoclasts. Therefore, we further evaluated the impact of US+HAMA@Cu(II)NS‐SPA on osteogenic, osteoclastic, and inflammatory markers around the implant. First, as shown in Figure 7A,E, there was a significant increase in the osteogenic marker OCN and the osteoclast inhibitor OPG in the US+HAMA@Cu(II)NS‐SPA group compared to other treatment groups. This suggests that US+HAMA@Cu(II)NS‐SPA can maintain periprosthetic bone homeostasis, promoting new bone formation and reducing bone destruction. Further analysis of inflammatory factor expression in tissues around the implant revealed that US+HAMA@Cu(II)NS‐SPA significantly reduced the expression of IL‐1β (Figure 7B,F), IL‐6 (Figure 7C,G), TNF‐α (Figure 7D,H), and other inflammatory factors. Consistent with the analysis of immunofluorescence, US+HAMA@Cu(II)NS‐SPA significantly reduced the expression of inflammatory factors and regulated growth factors related to osteogenesis and osteoclasts, promoting osteogenesis, alleviating bone destruction, and maintaining bone homeostasis. In summary, US+HAMA@Cu(II)NS‐SPA plays a highly significant role in the treatment of PJI.

As an innovative treatment strategy for intracellular infections in PJI, the biocompatibility of the therapeutic system is an essential consideration. Therefore, we conducted blood analysis and histological examinations on mice subjected to the treatment to assess potential systemic toxic side effects of the biomaterial system and its degradation products. After up to two weeks of treatment, the hematological and histological results of the US+HAMA@Cu(II)NS‐SPA group showed almost no differences compared to the control group, displaying no apparent systemic toxic side effects (Figure S9, Supporting Information). These findings indicate that the HAMA@Cu(II)NS‐SPA treatment system possesses excellent biocompatibility, providing a reference for its clinical application.

## 3 Conclusion

To summarize, this study presents an innovative bioorthogonal activation strategy, internally and externally mediated, utilizing micro/nano hydrogel microspheres. This approach is designed to enable efficient targeting and accurate delivery in the realm of SDT, ensuring enhanced therapeutic precision and efficacy. With surface modification by SPA, Cu(II)NS‐SPA is endowed with the ability to target and bind MRSA, enabling bacterial localization and tracking. Once bound to MRSA, Cu(II)NS‐SPA is efficiently phagocytosed by M*Φ* and activated intracellularly by BSH specifically secreted by MRSA, restoring its US responsiveness. Eventually, under the action of external US, it releases a large amount of ROS, precisely clearing intracellular bacteria. The experiments results demonstrate that this bioorthogonal activation strategy with micro/nano hydrogel microspheres effectively clears intracellular MRSA and significantly controls the progression of inflammation around implants and bone remodeling. Therefore, in clinical applications, we need to further develop ultrasound probes suitable for sonodynamic therapy of human bones and joints, as this is one of the major factors limiting this SDT strategy. Moreover, the hydrogel microspheres facilitate sustainable, precise SDT for up to two weeks, reducing intra‐articular injections while enhancing the bioavailability of the nanoparticles.

Of course, this treatment strategy still has some shortcomings before actual clinical application. For example, the difference in hardness between humans and mice is large, so stronger and more stable non‐attenuated ultrasound is needed to penetrate the bone and activate the sonosensitizer. Therefore, in clinical applications, we need to further develop ultrasound probes suitable for sonodynamic therapy of human bones and joints. In addition, in clinical applications, most doctors prefer high‐intensity focused ultrasound (HIFU) for the treatment of diseases. Therefore, if the activation of micro‐nano hydrogel microspheres can be achieved based on traditional HIFU equipment, it may greatly promote the clinical application of this study. Moreover, the adverse reactions and side effects of sonosensitizer at effective human doses are still unclear, which may further limit their application.

Overall, exemplified by PJI treatment, the application of these internally and externally mediated bioorthogonal activation micro/nano hydrogel microspheres offers a safe and effective solution to the clinical challenges of intracellular infections by multi‐drug resistant bacteria. With the continuous development of biomaterials, clinical transformation will eventually be achieved.

## 4 Experimental Section

### Materials

Copper(II) chloride (CuCl_2_), tetracarboxyphenylporphyrin (TCPP), 8‐arm‐PEG‐NH_2_, methacrylic anhydride (MA), hyaluronic acid (HA), 1,3‐Diphenylisobenzofuran (DPBF), 1‐(3‐Dimethylaminopropyl)‐3‐ethylcarbodiimide methiodide (EDC), N‐hydroxysuccinimide (NHS), *N*‐acryloxysuccinimide (NHS‐Ac), phosphate‐buffered saline (PBS), HEPES buffer solution, and other chemical reagents were acquired from Sigma Aldrich (USA), Maclin (China), and TCI (Japan). PD‐10 columns were purchased from GE Healthcare. Dialysis bags (Mw: 14 000) were obtained from Viskase. DCFH‐DA assay kit, Cell Counting Kit‐8 (CCK‐8), DAPI staining kit, DIR cell membrane staining kit, BSH‐BSSB assay kit were sourced from Beyotime Biotechnology. ELISA kits for OCN, OPN, OSM, RANKL, OPG, TGF‐β, IFN‐γ, TNF‐α, IL‐1β, IL‐2, IL‐6, etc., were obtained from Abcam (UK) and Cell Signaling Technology (USA). Staphylococcal Protein A antibody and MRSA strain *USA300* were procured from the National Engineering Research Center of Immunological Products.

### Preparation of Cu(II)NS‐SPA

We prepared a chelate of Cu and PEGylated TCPP following the method previously reported by our research group.^[^ ^16b^ ^]^ Specifically, 20 mg of TCPP, 400 mg of 8‐arm‐PEG‐NH_2_, and 10 mg of EDC were dissolved in 100 mL DMSO and stirred continuously for 24 h to ensure a complete reaction. The reaction mixture was then placed in a dialysis bag (molecular weight cutoff, 2000 Da) and dialyzed in 2 L water for 14 days (replace every two days) to collect the product, TCPP‐PEG. Subsequently, 134.45 mg of CuCl_2_ was dissolved in 1 mL of NaAc/HAc buffer (pH 5.5, 0.1 m Ac‐) and set aside. Equal volumes of the CuCl_2_ solution and TCPP‐PEG solution were thoroughly mixed and agitated at 70 °C for 180 min. The Cu(II)NS was then separated from the reaction solution using a PD‐10 column. SPA, possessing free thiol groups, can undergo a Michael addition reaction with carbon–carbon double bonds to achieve functionalization.^[^ ^24^ ^]^ Therefore, we first acryloylated Cu(II)NS to facilitate subsequent SPA modification. The specific steps were as follows: 30 mg of the previously synthesized Cu(II)NS was reacted with 10 mg of NHS‐Ac in HEPES buffer solution (10 mm HEPES, 150 mm NaCl, 10 mm EDTA) for 12 h, followed by separation of the acryloylated Cu(II)NS using a PD‐10 column. In the end, SPA was reacted with the separated product for 12 h at 4 °C, collected, and separated using a PD‐10 column to obtain the final product, Cu(II)NS‐SPA, which was stored at low temperature for future use. Next, we used a BSH assay kit to evaluate the reaction capability of Cu(II)NS and Cu(II)NS‐SPA with BSH, thus obtaining Cu(I)NS and Cu(I)NS‐SPA. More specifically, we mixed 10 mm Cu(II)NS‐SPA with 10 mm BSH solution(v/v = 1/2), shaken at 37 °C for 2 h, Cu(I)NS‐SPA was then separated using a PD‐10 column. Cu(I)NS was obtained using a similar method. Note that all operations should be conducted in a dark environment.

### Characterization of Cu(II)NS‐SPA

A Zetasizer (Malvern Nano‐ZS, UK) was used to determine the size and Zeta potential of Cu(II)NS and Cu(II)NS‐SPA nanoparticles. The Zetasizer microsample cell was first rinsed alternately with distilled water and anhydrous ethanol. Then, 10 µl of the nanoparticle sample was diluted to 1 mL with distilled water and placed into the microsample cell for dynamic light scattering analysis. Furthermore, to assess the molecular structure and properties of Cu(II)NS‐SPA, we measured the UV absorption spectra of TCPP‐PEG, Cu(II)NS, and Cu(II)NS‐SPA using a UV–vis spectrophotometer (PE lambda 750, USA). For TEM evaluation, samples were equilibrated at room temperature for 0.5 h. The sample (5 µl) was dropped onto an ultra‐thin carbon film and left to settle for 5 min. Excess liquid was removed. Subsequently, the FEI Tecnai F20 instrument was used to observe the nanoparticle morphology.

### Detection of ROS in Solution

This study utilized the degradation of DPBF as a measure to evaluate the efficiency of ROS production by nanoparticles. To validate that Cu(II)NS‐SPA, once chelated with Cu^2+^, lacks sonosensitivity but can generate ROS under US activation after reduction by BSH, the following procedure was conducted. To start off, 100 µl of Cu(I)NS‐SPA (10 µm Cu) and 100 µl of Cu(II)NS‐SPA (10 µm Cu) were each thoroughly mixed with 2 mL of DPBF solution (200 µm). After US (2 W cm^−2^, 1 MHz, 50% duty cycle) treatment for 0, 30, 60, 90, 120, 150, and 180 s, the DPBF degradation degree was calculated by measuring the solution's absorbance at 415 nm using a UV–vis spectrophotometer. Following this, we further assessed the impact of different US treatment intensities and durations on the rate of ROS production. Cu(I)NS‐SPA (10 µm Cu; 100 µl) was thoroughly mixed with 2 mL of DPBF solution (200 µm). The mixture was then treated with US at powers of control, 0.5, 1, 1.5, and 2 W cm^−2^ (1 MHz, 50% duty cycle) for durations of 0, 30, 60, 90, 120, 150, and 180 s. The degradation of DPBF was then measured and calculated using a UV–vis spectrophotometer. US treatment were conducted by Chattanooga US transducer. The effective area of the ultrasonic probe was 2 cm^2^. Before treatment, an ultrasonic coupling agent (1 g) was placed on the ultrasonic probe. The intensity was determined by the instrument output. To ensure the accuracy of the output, the manufacturer will calibrate this instrument each year.

### Preparation of HAMA@Cu(II)NS‐SPA

Initially, previously reported methods were used for synthesizing HAMA.^[^ ^25^ ^]^ A 500 mL solution of hyaluronic acid (HA) (2 wt.%) was prepared and slowly supplemented with 20 mL of methacrylic anhydride (MA). Then, 20 mL of NaOH (5 m) was added dropwise using a microsyringe pump, and the reaction was carried out overnight under ice bath conditions. After completion, Dialysis was performed in deionized water for three days to purify the reaction mixture (molecular weight cutoff, 3500 Da). The purified product was then freeze‐dried and stored at −20 °C for future use. The grafting rate of MA was determined by 1H NMR (600 MHz, Bruker, Germany). Microfluidic technology was employed to prepare hydrogel microspheres loaded with Cu(II)NS‐SPA. Specifically, a solution containing 5 wt.% HAMA, 1 wt.% Cu(II)NS‐SPA and 0.5 wt.% photoinitiator was injected into a microfluidic oil phase composed of Span 80 and 95 wt.% paraffin oil, forming pre‐gel droplets. The droplets were collected in a low‐temperature environment and cured with UV light. The size of the microspheres was controlled by adjusting the flow rate ratio of the aqueous solution to the oil phase. The UV‐cured hydrogel microspheres were then sequentially washed with anhydrous ether and deionized water to remove the oil phase and stored in deionized water at 4 °C for future use. HAMA hydrogel microspheres were prepared using a similar method. Using a bright field microscope(LSM800, ZEISS, Germany), we observed the morphology and size of pre‐gel droplets and UV‐cured microspheres. For morphological observation, critical point drying was performed, and the HAMA@Cu(II)NS‐SPA and HAMA microspheres were viewed using SEM (FEI Sirion 200, USA). To confirm the successful incorporation of Cu(II)NS‐SPA into HAMA microspheres, Cu(II)NS‐SPA was first labeled with Cy3, then hydrogel microspheres were fabricated with HAMA following the previously described procedure. Finally, the Cy3‐labeled Cu(II)NS‐SPA within the hydrogel microspheres was observed under laser scanning confocal microscopy (LSCM).

### Degradation Assessment and Drug Release Analysis

We quantified the degradation of HAMA@Cu(II)NS‐SPA employing a method outlined in the existing literature.^[^ ^25^ ^]^ A quantity of 5 mg of hyaluronidase was incorporated into a 1 mL dispersion of HAMA@Cu(II)NS‐SPA microspheres (3 wt.%, pH = 7.4), which was then subjected to continuous agitation at 37 °C. The hyaluronidase solution was refreshed bi‐daily. The rate of residue remaining in the samples was meticulously recorded at specifically designated time intervals. To investigate the release dynamics of HAMA@Cu(II)NS‐SPA, we immersed it in a 1 mL, 0.1% bovine serum albumin solution, sustained in a 37 °C constant temperature environment. The concentration of Cu(II)NS‐SPA in the supernatant was determined at set time points using UV spectrophotometry.

### MIC Determination

We inoculated the MRSA strain *USA300*, which was stored at −80 °C in glycerol stock, on Tryptic Soy Agar (TSA) plates and cultured for 24 h at a constant temperature. A colony was then selected and dissolved in 2 mL of Tryptic Soy Broth (TSB), followed by re‐inoculation onto TSA plates. This process was repeated 2–3 times until an active colony was selected and dissolved in 5 mL of TSB until OD600 reached 0.5. To exclude the influence of US on bacterial growth, 1 mL of diluted bacteria solution was added into Eppendorf tubes, each treated with US at powers of control, 0.5, 1, 1.5, and 2 W cm^−2^ (1 MHz, 50% duty cycle) for durations of 0, 30, 60, 90, 120, 150, and 180 s, and then incubated for 18 h at 37 °C. Subsequently, Cu(II)NS‐SPA and Cu(I)NS‐SPA at concentrations of 0, 0.5, 1.0, 2.0, 5.0, and 10.0 µm were added to the diluted bacterial solution, treated with the US at a power of 2 W cm^−2^ (1 MHz, 50% duty cycle) for 2 min, and incubated at 37 °C for 18 h before measuring the OD_600_ of the bacterial solution.

### Activation of Cu(II)NS‐SPA by BSH

To simulate the expression of BSH by MRSA within M*Φ*, we infected RAW 264.7 cells with MRSA for 2 h, using a multiplicity of infection (MOI) of 10. Subsequently, extracellular MRSA was eradicated by gentamicin, followed by washing the macrophages with a serum‐free medium. Cells were then lysed in a solution containing 0.1% Triton‐X in Hb, which lysed the macrophages without damaging intracellular bacteria. The lysates were serially diluted in a PBS solution containing 0.05% Tween‐20, which was employed to disrupt bacterial aggregates, resulting in the acquisition of MRSA with a high secretion state of BSH. Subsequently, the treated MRSA was incubated with Cu(II)NS‐SPA at 37 °C. At specified time points, the content of BSSB was measured using a BSSB assay kit, and the production of Cu(I)NS‐SPA was detected using a Cu^+^ ion probe. Additionally, US stimulation was applied at designated time points, followed by measuring the OD600 of the bacterial solution after 18 hours of incubation, to assess the bactericidal capability of Cu(II)NS‐SPA under the bioorthogonal stimulation formed by BSH and US. Furthermore, extracts of HAMA@Cu(II)NS‐SPA, Cu(II)NS‐SPA, HAMA, and saline were incubated with the bacterial solution, with and without US stimulation. Standard colony counting was then employed to evaluate the antimicrobial activity of HAMA@Cu(II)NS‐SPA against MRSA.

### Bacterial Live and Dead Staining

A 1 mL sample of bacterial suspension was incubated with an equal volume of extract from HAMA@Cu(II)NS‐SPA, Cu(II)NS‐SPA, HAMA, and PBS, both with and without US treatment. After incubation, the bacterial suspensions were stained with a mixture of SYTO 9 and propidium iodide (PI) and incubated in the dark for 0.5 h at 37 °C. A bacterial solution (10 µl) was placed on a slide and observed using LSCM.

### Detection of Reactive Oxygen Species in Bacteria

Bacteria treated with nanoparticles and US were assessed for ^1^O_2_ generation using a ROS detection assay kit. Briefly, the DCFH‐DA probe was added to 1 mL of diluted bacterial suspension and incubated for 20 min at 37 °C. Post‐incubation, unbound probes were washed off with PBS, and an equal volume of extract from HAMA@Cu(II)NS‐SPA, Cu(II)NS‐SPA, HAMA, or PBS was added, with or without US treatment. Singlet oxygen within the bacteria oxidizes DCFH to form fluorescent DCF, which emits green fluorescence under 480 nm excitation. Following washes with PBS, 10 µl of the bacterial solution was placed on a slide, and observed using LSCM.

### Antibiotic Resistance Testing

Using the above protocol and starting with the original bacterial strain (Generation 0), MRSA was subjected to OD_600_ measurement. To be brief, MRSA bacterial suspensions treated with Cu(I)NS‐SPA and US (OD_600 _= 0.5) were cultured at 37 °C for 18 h before OD_600_ of the bacterial suspension was measured. Next, after culturing on TSA plates for 24 h, a single bacterial colony (representing surviving cells from the previous SDT treatment) was carefully selected from the plate and identified as Generation 1 cells. Generation 1 bacterial colonies were dissolved in 2 mL TSB, cultured until OD600 = 0.5, subjected to SDT, and used for the next round of OD_600_ measurement. This process was repeated to assess the impact of SDT on MRSA over 10 consecutive generations.

### Biofilm Inhibition and Disruption Tests

The experimental groups were designed as previously described. F Biofilm inhibition experiments were conducted using polystyrene 96‐well plates, with each well containing 100 mL of nanoparticle suspension and 100 mL of bacterial suspension. In the US treatment groups, bacterial suspensions were treated immediately after adding nanoparticles. Following a static incubation at 37 °C for 24 h, the culture medium was gently discarded. Each well was then washed twice using 100 µL of PBS and air‐dried for 30 min at ambient temperature. The biofilms were fixed with 100 µL of anhydrous ethanol for another 30 min at room temperature. Post‐fixation, wells were air‐dried and stained with 100 µL of 0.1% (v/v) crystal violet for 20 min. After staining, the wells were rinsed with PBS until the runoff was clear, air‐dried, and photographed to assess biofilm formation. For quantitative analysis, 200 µL of anhydrous ethanol was added to each well to solubilize the bound dye. The wells were then incubated at 37 °C for 30 min to facilitate elution. The optical density of the resulting solution was measured at a wavelength of 590 nm to determine the biofilm biomass. For biofilm disruption tests, stable biofilms formed by static bacterial culture for 24 h were subjected to US treatment. Then, the treated biofilms were incubated at a constant temperature for another 24 h, followed by crystal violet staining and observation as described above.

### SEM Observation of Bacteria

The experimental groups were designed as previously described. Bacterial sediment samples post‐treatment were collected in 1.5 mL Eppendorf (EP) tubes, to which 1.2 mL of pre‐chilled electron microscopy fixative (2.5% glutaraldehyde in phosphate‐buffered saline, pH = 7.2) was added slowly. The samples were initially stored at 4 °C overnight. The following day, they underwent a series of washes—three 15‐minute rinses with phosphate‐buffered saline (PBS). Fixation was then achieved by treating the samples with a 1% osmium tetroxide solution for two hours. Subsequent to fixation, the samples were subjected to further rinses with PBS before a dehydration process using an ascending ethanol series (30%, 50%, 70%, 80%, 90%, and 95%) was initiated. This step was completed with two 20‐minute immersions in anhydrous ethanol. The samples were treated with an equal‐volume mixture of ethanol and isoamyl acetate for 30 min, followed by pure isoamyl acetate for one hour. Upon completing the critical point drying process, the samples were coated and then examined with SEM.

### Detection of Bacterial Protein and Nucleic Acid Leakage

The experimental groups were set up as described previously. The supernatant of the treated bacterial solution was collected, and its absorbance at 260 nm was measured to assess nucleic acid leakage. The amount of leaked protein was detected using a BCA Protein Assay Kit (Thermo Scientific Cat. No. 23 225), and the bovine serum albumin standard was used to calibrate the BCA method. Briefly, the prepared BCA working solution was mixed with the supernatant at a 10:1 ratio. An absorbance measurement at 562 nm was performed after 0.5 h of incubation at 37 °C.

### Cell Biocompatibility

RAW264.7 cells and BMMs were used for cell biocompatibility testing. Dulbecco's Modified Eagle Medium (DMEM) containing 10% fetal bovine serum (FBS) and 1% penicillin‐streptomycin was used for the culture of RAW264.7 cells. BMMs were derived from C57BL/6 mice. Specifically, after euthanizing the mice and performing thorough disinfection, femurs and tibias were transferred to a biosafety cabinet and placed in PBS containing 1% penicillin‐streptomycin. Soft tissues such as muscles were meticulously removed, and the bones were transferred to a new dish. The ends of the femurs and tibias were cut, and the bone marrow was thoroughly expelled using a syringe filled with culture medium. The cell clumps were dispersed by repeated pipetting, and We filtered the cells by a 70 µm cell strainer. After centrifugation at 2500 rpm min^−1^ for 5 min, the supernatant was discarded, and cells were resuspended in BMMs induction medium and plated. Cells were cultured for 7 days before being used for subsequent experiments. The Cell Counting Kit‐8 (CCK‐8) was used to determine the viability of BMMs and RAW264.7 cells. Specifically, BMMs and RAW264.7 cells were co‐cultured with Cu(II)NS‐SPA at concentrations of 0, 0.5, 1.0, 2.0, 5.0, and 10.0 µm for 24, 48, and 72 h. After washing three times with PBS at designated time points, incubation continued for an additional two hours with DMEM containing CCK‐8 solution (1:10 v/v). Microplate readers (Bio‐Rad 680, USA) were used to measure the absorbance at 450 nm for each well. The effects of HAMA@Cu(II)NS‐SPA, Cu(II)NS‐SPA, and HAMA on cell viability were assessed analogously. Live/dead staining was also used to evaluate the cytotoxicity of HAMA@Cu(II)NS‐SPA, Cu(II)NS‐SPA, and HAMA. To be more precise, HAMA@Cu(II)NS‐SPA, Cu(II)NS‐SPA, HAMA, and PBS were co‐cultured with BMMs and RAW264.7 cells for 24, 48, and 72 h. At specific time points, the number of live and dead cells was determined using LSCM after fluorescent dyes were added to a LIVE/DEAD staining kit.

### Intracellular Bacterial Clearance Capability

To visually monitor the growth of MRSA, that tagged with green fluorescent protein (GFP) was constructed using a homologous recombination method.^[^ ^18b^ ^]^ MRSA cultured to exponential growth phase was incubated with Cy3‐labeled HAMA@Cu(II)NS‐SPA extract for 1 h so that Cu(II) NS‐SPA could fully combine with MRSA and exert its opsonizing effect. And then used to infect RAW 264.7 cells for 2 h with a multiplicity of infection (MOI) of 10. The control group directly used exponentially growing MRSA to infect RAW 264.7 cells for 3 h. The experimental group was further treated with US and gentamicin to clear both intracellular and extracellular MRSA. Subsequently, the cell membrane was stained with DIR fluorescent dye as per the operational guidelines, and the number of intracellular and extracellular MRSA was observed using LSCM. Finally, the supernatant and lysis fluid were subjected to standard colony counting to evaluate the growth of intracellular and extracellular MRSA.

### Construction of PJI Animal Model

All animal experiments were approved by the Animal Research Committee of Chongqing Medical University Laboratory Animal Center (The Ethical Clearance number is IACUC‐CQMU‐2023‐11033). In animal experiments, male rats are the most commonly used animals for bone and joint‐related diseases, because male rats have a larger body size than female rats, which is more conducive to the construction of PJI models.^[^ ^26^ ^]^ In addition, there is no clear evidence that gender has a significant impact on the results of PJI animal models. So, we chose male rats as the research subjects. The Chongqing Medical University Laboratory Animal Center provided adult Sprague‐Dawley rats for in vivo PJI studies. The rats were anesthetized with 2% isoflurane inhalation, and after disinfection and draping, an incision was made on the medial side of the knee joint. The quadriceps‐patella complex was laterally retracted to fully expose the distal femur. Using a dental drill with a 0.8 mm diameter bit, the bone marrow cavity was opened and expanded at the center point of the femoral condyle. To establish the PJI model, 10 µl of MRSA culture (1 × 10^5^ CFU mL^−1^) was slowly and carefully injected into the marrow cavity. Subsequently, a 1.1 × 10 mm titanium alloy screw was slowly implanted, the patella repositioned, and the incision sutured layer by layer. Animals were divided into Control, Cu(II)NS‐SPA, HAMA@Cu(II)NS‐SPA, US+Cu(II)NS‐SPA, and US+HAMA@Cu(II)NS‐SPA groups. Intrarticular injections of PBS, Cu(II)NS‐SPA, and HAMA@Cu(II)NS‐SPA were then administered according to group allocation. The US+Cu(II)NS‐SPA and US+HAMA@Cu(II)NS‐SPA groups received daily US stimulation at a power of 2 W cm^−2^ (1 MHz, 50% duty cycle, 2 min). The US+Cu(II)NS‐SPA group required daily intra‐articular injections, whereas the HAMA@Cu(II)NS‐SPA group only underwent a single intra‐articular administration. The rats were euthanized after 2 weeks, the skin of the joint cavity incised, and local abscesses photographed for observation. The implants were removed and sonicated in 2 mL PBS for 5 min. The detached bacteria were diluted, inoculated onto LB medium, incubated at 37 °C for one day, and bacterial CFUs counted.

### Radiological Evaluation

X‐ray examinations of the excised rat femoral tissues were performed using an X‐ray machine (Faxitron, USA) to assess bone resorption. Subsequently, the excised rat femoral tissues were further analyzed using micro‐CT (SCANCO, Switzerland). 3D images of the femur and periprosthetic trabeculae were obtained, and quantitative analyses were conducted on parameters such as Tb.N, BMD, BV, BV/TV, and bone surface area to bone volume ratio (BS/BV).

### Histological Evaluation

Rat femurs were obtained and fixed in 4% paraformaldehyde solution, followed by decalcification in 10% EDTA solution for one month, with the EDTA solution changed every three days. After decalcification, the tissues were embedded in paraffin, and 5 µm thick pathological sections were prepared for subsequent analysis. Masson's trichrome, Hematoxylin and eosin (H&E), Gram, and TRAP staining were used to assess inflammatory infiltration, new bone formation, bacterial residue, and osteoclast activity around the implant. For immunofluorescence staining, sections were treated with 5% BSA solution at 37 °C for 1 h. They were then incubated overnight at 4 °C with rabbit polyclonal antibodies against OCN, OPG, IL‐1β, IL‐6, and TNF‐α. After washing with TBST, the sections were exposed to fluorescence‐labeled secondary antibodies for 1 h, washed with buffer solution, and observed using LSCM. Quantitative analysis of the expression levels of OCN, OPG, IL‐1β, IL‐6, and TNF‐α was conducted using ImageJ software.

### ELISA Testing

Tissue samples from around the implant were collected, homogenized in saline, and centrifuged at 1000 g min^−1^ for 10 min. Supernatants were then stored at 4 °C for further analysis. In order to detect the expression of factors (including TGF‐β, TNF‐α, IFN‐γ, IL‐6, IL‐2, IL‐1β, OCN, OPN, OPG, and RANKL,), ELISA kits were used according to the manufacturer's instructions.

### Biocompatibility

To study the biocompatibility of HAMA@Cu(II)NS‐SPA, 200 µl of blood was collected from the orbital venous plexus for hematological analysis two weeks post‐surgery, and the mice were then euthanized. Histological analysis of the heart, liver, spleen, lungs, and kidney was conducted using paraformaldehyde‐fixed sections of the organs.

### Statistical Analysis

Error bars represent the standard deviation of the independent sample mean. A comparison of experimental data between the two groups was performed using the student‐*t* test. Use one‐way or two‐way analysis of variance (ANOVA) to compare experimental data between multiple groups. For materials science‐related experiments, data were derived from five technical replicates. For in vitro bacteria and cell‐related experiments, the data come from five biological replicates. For animal experiments, in view of the high cost and the stability of animal experiment results, we used three animal replicates. All statistical analyzes were performed using GraphPad Prism 9.5.0. The significance of differences was defined as *P < 0.05, **P < 0.01, ***P < 0.001, and ****P < 0.0001. NS represents no statistical difference.

## Conflict of Interest

The authors declare no conflict of interest.

## Supporting information



Supporting Information

## Data Availability

The data that support the findings of this study are available from the corresponding author upon reasonable request.
