# Supplementary material for: Precise Clearance of Intracellular MRSA via Internally and Externally Mediated Bioorthogonal Activation of Micro/Nano Hydrogel Microspheres
Source: Adv Sci (Weinh). 2024 Sep 29;11(44):2402370. doi: 10.1002/advs.202402370 (PMC11600240; doi:10.1002/advs.202402370)
Supplement: Supplementary file 1 — Supporting Information [file ADVS-11-2402370-s001.docx]

**Precise Clearance of Intracellular MRSA via Internally and Externally Mediated Bioorthogonal Activation of Micro/Nano Hydrogel Microspheres**

*Jianye Yang, Li Chen, Zhengwei Cai, Libin Pang, Yanran Huang, Pengcheng Xiao, Juan Wang, Wei Huang*, Wenguo Cui*, Ning Hu**

J. Yang, L. Chen, Y. Huang, P. Xiao, Prof. W. Huang, Prof. N. Hu

Department of Orthopaedics, The First Affiliated Hospital of Chongqing Medical University, Orthopedic Laboratory of Chongqing Medical University, Chongqing 400016, China

E-mail: huncqjoint@yeah.net (N. Hu), huangwei68@263.net (W. Huang)

Z. Cai, L. Pang, J. Wang, Prof. W. Cui

Department of Orthopaedics, Shanghai Key Laboratory for Prevention and Treatment of Bone and Joint Diseases, Shanghai Institute of Traumatology and Orthopaedics, Ruijin Hospital, Shanghai Jiao Tong University School of Medicine, 197 Ruijin 2nd Road, Shanghai 200025, P. R. China.

E-mail: wgcui80@hotmail.com (W.G. Cui)

*Corresponding author.


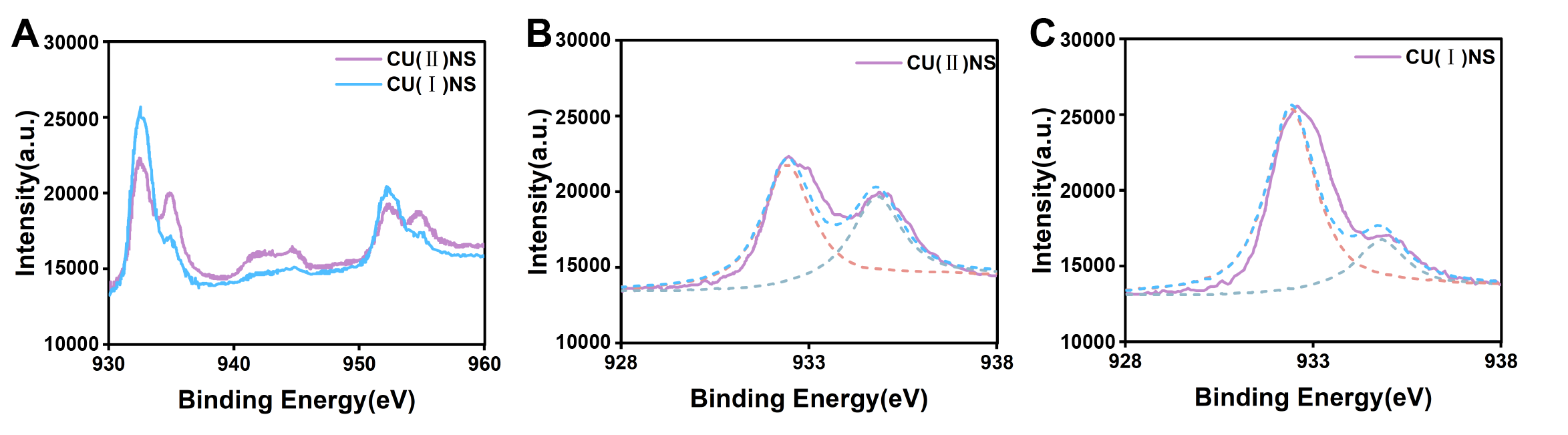


**Figure S1.** XPS of Cu(II)NS and Cu(I)NS. (A) XPS spectra (Cu 2p) of Cu(II)NS and Cu(I)NS. (B, C) XPS peak fitting of Cu(II)NS (B) and of Cu(I)NS (C).

**
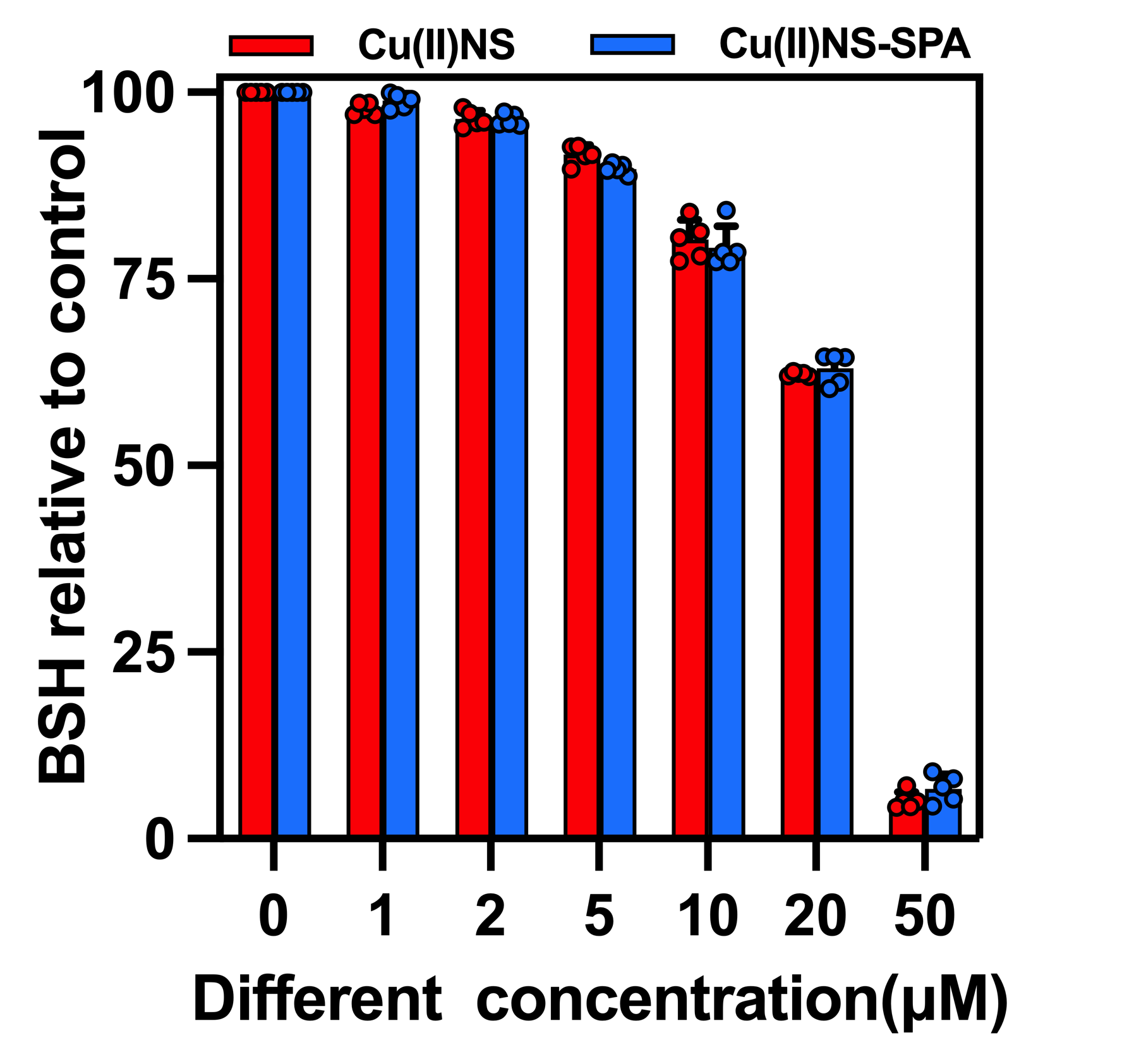
**

**Figure S2.** Reactivity of Cu(II)NS and Cu(II)NS-SPA with BSH.


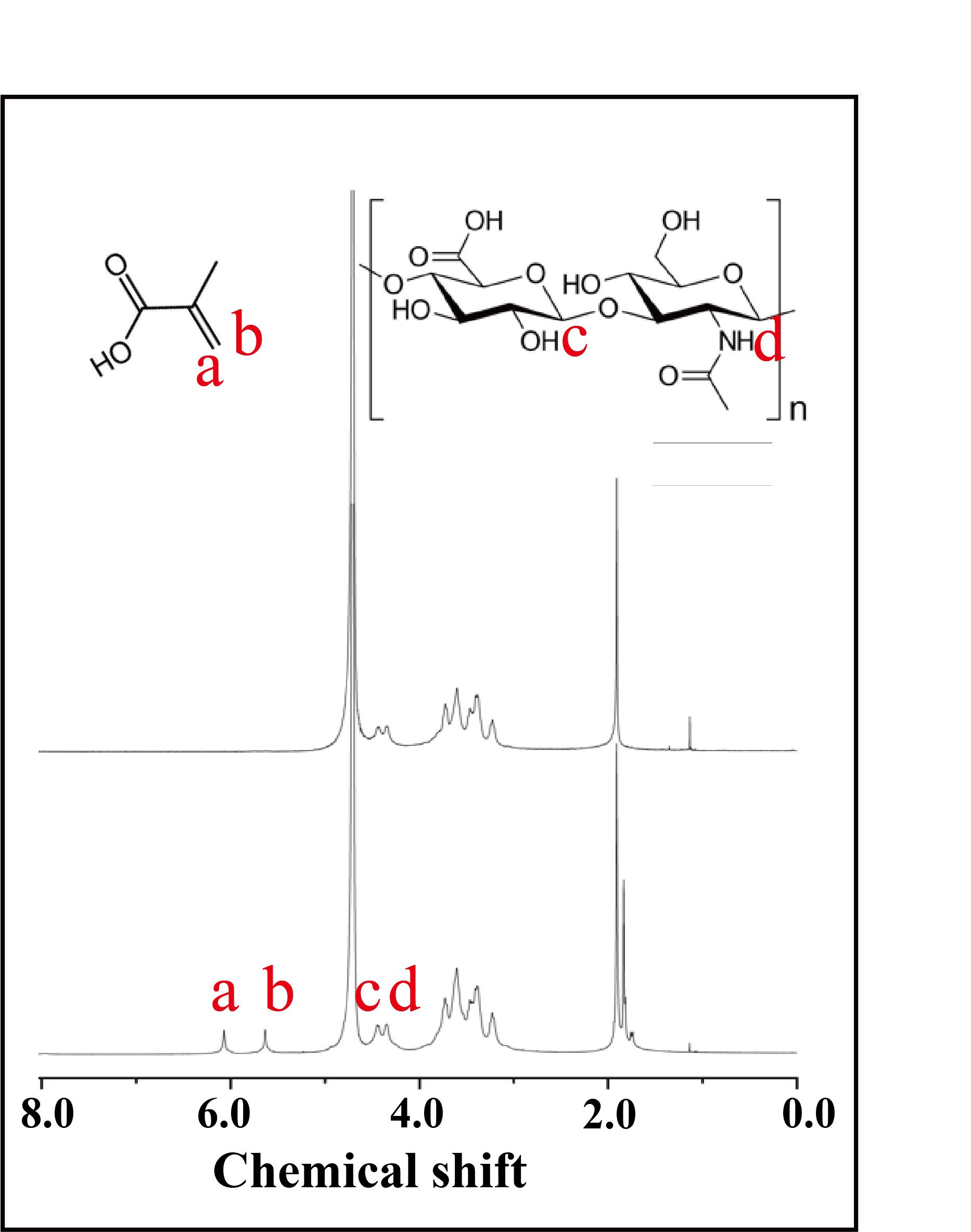


**Figure S3.** NMR hydrogen spectrum of methacrylated hyaluronic acid.


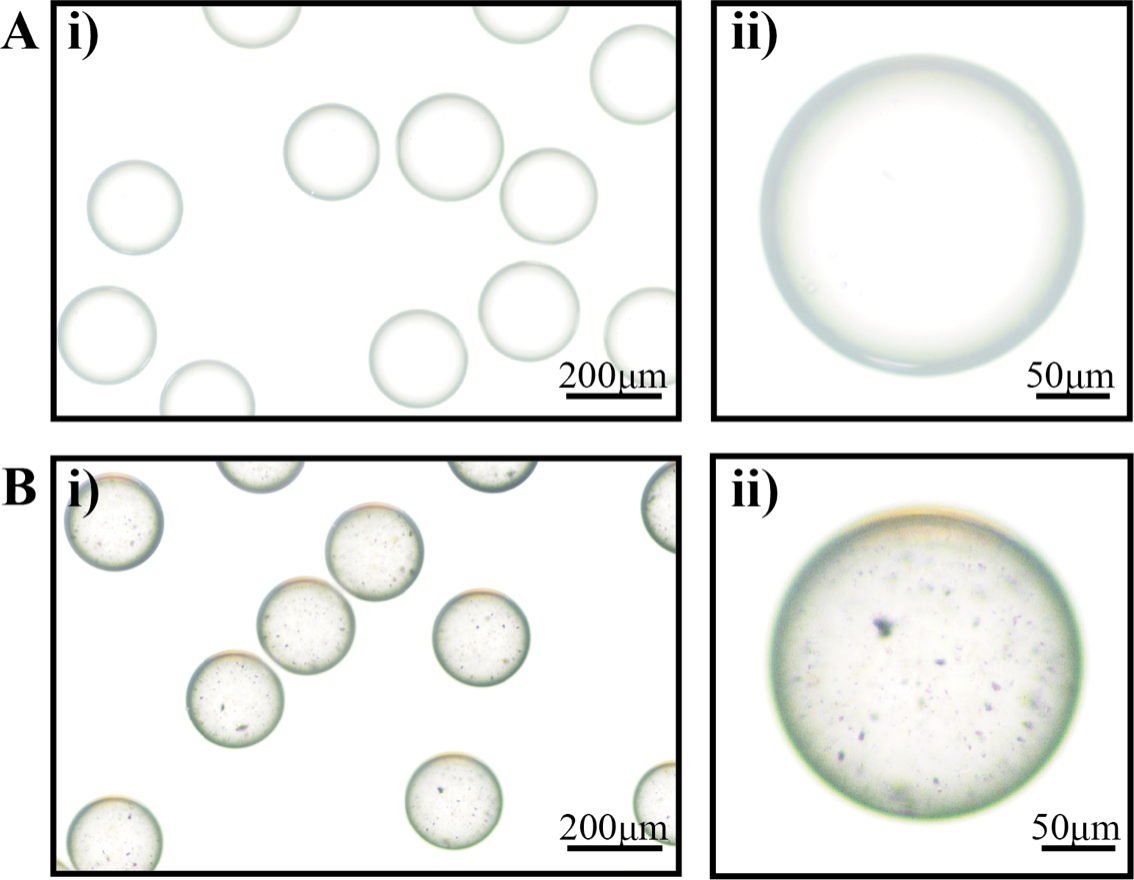


**Figure S4.** Bright field images of HAMA and HAMA@Cu(II)NS-SPA pre-gel droplets.


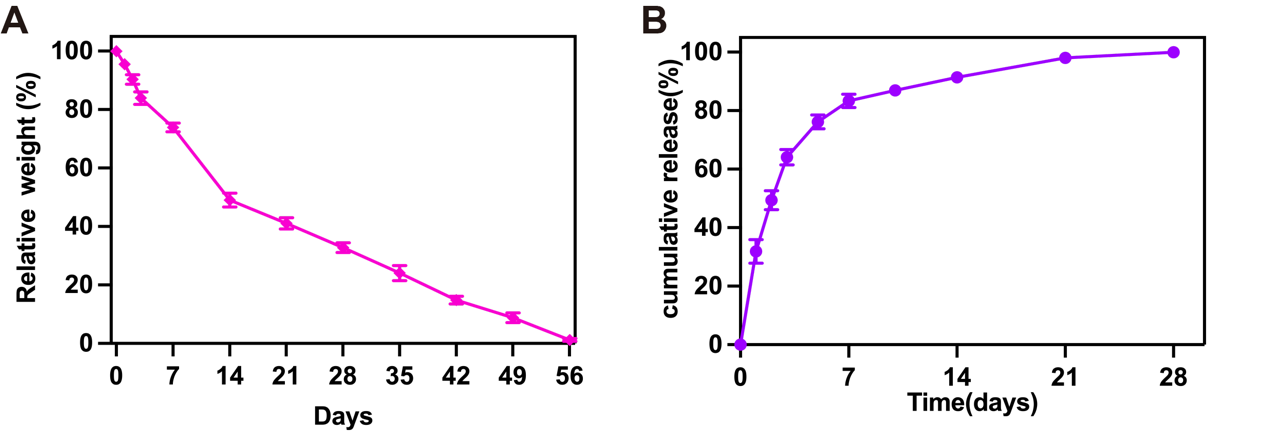


**Figure S5.** Degradation curve (A) and release curve (B) of HAMA@Cu(II)NS-SPA.

**Figure S6.** The effect of ultrasound treatment on bacterial growth at different intensities (A) and durations (B).


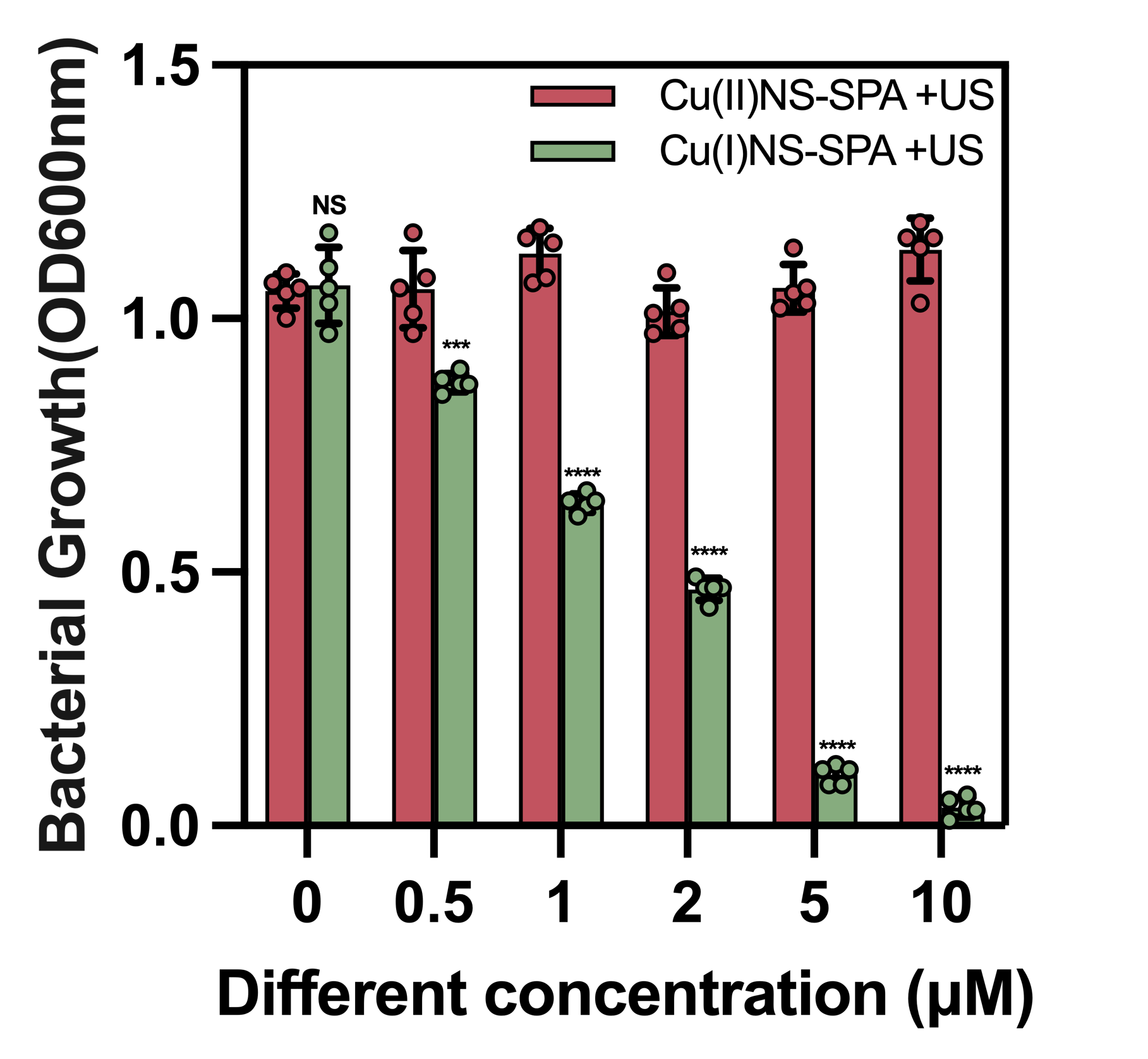


**Figure S7.** The effect of the same ultrasound treatment on bacterial growth for Cu(II)NS-SPA and Cu(I)NS-SPA.


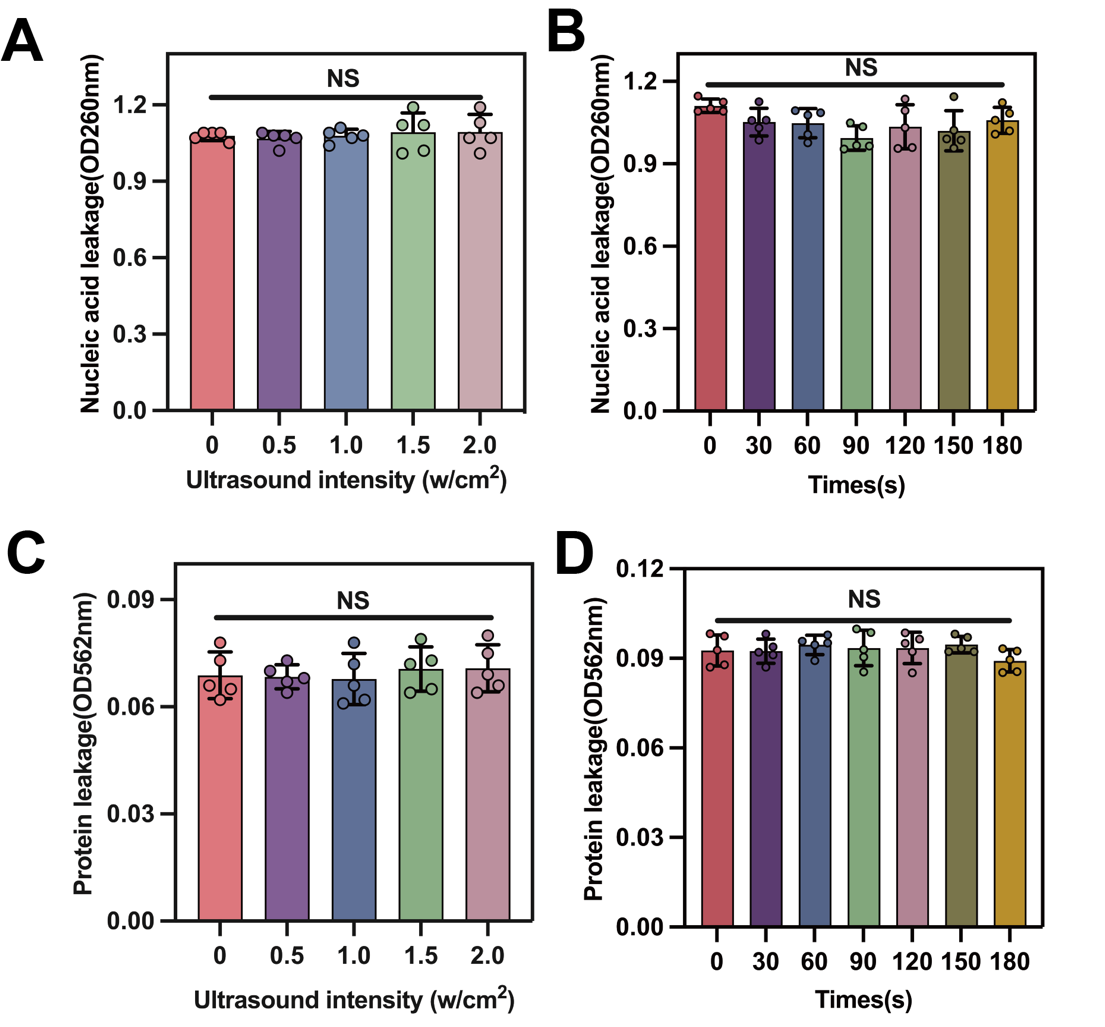


**Figure S8.** Ultrasound treatment alone does not affect the leakage of bacterial proteins and nucleic acids.


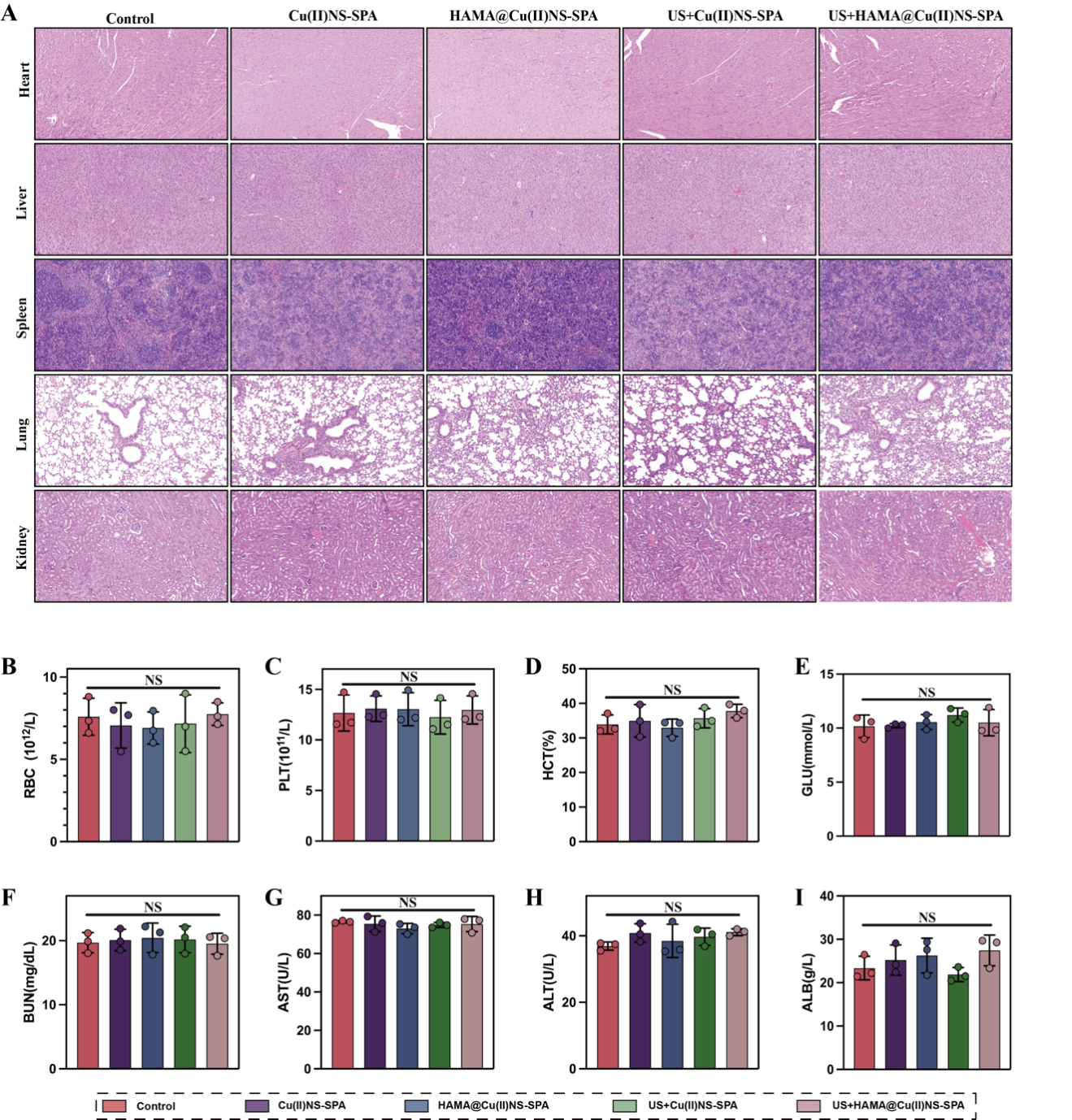


**Figure S9.** In vivo biocompatibility of biorthogonally activated hydrogel microspheres mediated internally and externally. (A) Representative HE-stained images of the heart, liver, spleen, lungs, and kidneys. (B-I) Complete blood count and biochemical analysis two weeks post-operation. RBC: red blood cells; PLT: platelets; HCT: hematocrit; GLU: glucose; BUN: blood urea nitrogen; AST: aspartate aminotransferase; ALT: alanine aminotransferase; ALB: albumin.
